# S100P contributes to promoter demethylation and transcriptional activation of SLC2A5 to promote metastasis in colorectal cancer

**DOI:** 10.1038/s41416-021-01306-z

**Published:** 2021-06-29

**Authors:** Mingdao Lin, Yuan Fang, Zhenkang Li, Yongsheng Li, Xiaochuang Feng, Yizhi Zhan, Yuwen Xie, Yuechen Liu, Zehao Liu, Guoxin Li, Zhiyong Shen, Haijun Deng

**Affiliations:** 1 Department of General Surgery, Nanfang Hospital, Southern Medical University, Guangzhou, Guangdong Province China; 2 Department of Radiation Oncology, Nanfang Hospital, Southern Medical University, Guangzhou, Guangdong Province China; 3 Department of Pathology, Nanfang Hospital, Southern Medical University, Guangzhou, Guangdong Province China

**Keywords:** Metastasis, Tumour biomarkers, Colorectal cancer

## Abstract

**Background:**

SLC2A5 is a high-affinity fructose transporter, which is frequently upregulated in multiple human malignant tumours. However, the function and molecular mechanism of SLC2A5 in colorectal cancer (CRC) remain unknown.

**Methods:**

We detected the expression levels of SLC2A5 in CRC tissues and CRC cell lines by western blotting, qRT-PCR and immunohistochemistry. CRC cell lines with stable overexpression or knockdown of SLC2A5 were constructed to evaluate the functional roles of SLC2A5 in vitro through conventional assays. An intrasplenic inoculation model was established in mice to investigate the effect of SLC2A5 in promoting metastasis in vivo. Methylation mass spectrometry sequencing, methylation specific PCR, bisulphite sequencing PCR, ChIP-qPCR and luciferase reporter assay were performed to investigate the molecular mechanism underlying transcriptional activation of SLC2A5.

**Results:**

We found that SLC2A5 was upregulated in colorectal tumour tissues. Functionally, a high level of SLC2A5 expression was associated with increased invasion and metastasis capacities of CRC cells both in vitro and in vivo. Mechanistically, we unveiled that S100P could integrate to a specific region of SLC2A5 promoter, thereby reducing its methylation levels and activating SLC2A5 transcription.

**Conclusions:**

Our results reveal a novel mechanism that S100P mediates the promoter demethylation and transcription activation of SLC2A5, thereby promoting the metastasis of CRC.

## Background

Colorectal cancer (CRC) is the third most common malignant tumour globally with approximately 1.8 million newly diagnosed cases and 860,000 deaths in 2018.^1^ In addition, the incidence and mortality of CRC are increasingly higher and tending to be younger.^2^ In recent years, despite the rapid progress in pathogenesis and treatment, recurrence and metastasis are still the main causes of death in patients with CRC.^3,4^ Therefore, understanding the complex molecular mechanisms driving tumour metastasis and finding effective prognostic and therapeutic targets are of vital importance.^3,4^

S100 calcium-binding protein P (S100P) is a small calcium-binding protein of S100 family with two EF hand calcium-binding motifs, involving in the regulation of signalling pathways, protein phosphorylation and cell proliferation and differentiation.^5,6^ Previous studies have shown that S100P was produced from the cytoplasm and then transported outside the cell by binding to Ezrin. The extracellular S100P further bound to the cell surface receptor RAGE, and then activated the downstream MAPK/ERK pathway to promote tumour proliferation and invasion.^7,8^ Numerous studies have shown that S100P was upregulated in diverse cancers and associated with poor clinical prognosis.^9–12^ However, previous studies have mostly described the mechanisms of exocrine S100P or its roles in cytoplasm. Based on our previous study underlying the mechanism of S100P in driving CRC metastasis, we found that S100P was also abundantly expressed in the nucleus.^13^ In consideration of the potentially transcriptional function of S100P, we performed ChIP-sequencing analysis and found that S100P could interact with multiple genes, in which the promoter of SLC2A5 gene showed the most obvious interaction with S100P. Therefore, we were interested in the roles of SLC2A5 in CRC.

Solute Carrier Family 2 Member 5 (SLC2A5) is a high-affinity fructose transporter, mainly expressed in the intestinal epithelial cells.^14–16^ It was originally reported that the high level of SLC2A5 expression was associated with hypertension and diabetes.^17,18^ Recent studies have shown that SLC2A5 was also involved in cancer processes.^19^ In breast cancer, increased expression of SLC2A5 promoted tumour cell proliferation, while knockdown of SLC2A5 significantly suppressed tumour growth.^20^ In lung adenocarcinoma, ectopic SLC2A5 expression significantly promoted tumour growth and distant metastasis.^21^ In addition, it has been reported that SLC2A5 was related to poor prognosis of patients with several cancers such as acute myeloid leukaemia, glioma and lung adenocarcinoma.^21–23^ Although the tumour-promoting role of SLC2A5 has been gradually revealed, in-depth mechanism investigation is still scarce. In addition, the biological function of SLC2A5 in CRC remains unknown.

DNA methylation is the most studied epigenetic modification, which is essential for facilitating vital biological processes such as embryonic development, genomic imprinting and X-chromosome inactivation.^24^ Normally, the methylation and demethylation of the genome maintain a dynamic equilibrium. Once this balance is disrupted, abnormal gene expression will occur, leading to various pathological conditions, including carcinogenesis.^25^ Promoter demethylation is commonly known to be an important approach for oncogene activation.^26^ However, the mechanism by which S100P regulates SLC2A5 promoter demethylation has never been reported to our knowledge.

Here, we evaluated the expression levels of SLC2A5 in CRC tissues and cell lines, and further elucidated the role and molecular mechanism of SLC2A5 in promoting tumour metastasis. Our findings provide a new insight into the epigenetic transcriptional activation of SLC2A5 mediated by S100P.

## Methods

### Patients and samples

The human CRC tissue samples used in this study were obtained from Department of General Surgery at Nanfang Hospital (Guangzhou, China), from 1 January 2013 to 1 January 2017, and approved by the Institute Research Medical Ethics Committee of Nanfang Hospital. A total of 214 specimens involving 178 patients were included in our analysis, including 36 pairs of tumour and matched normal mucosa samples, a tissue microarray (TMA) of 142 patients’ resections of colorectal tumour and distal normal mucosa, purchased from the National Engineering Center for Biochip at Shanghai. All patients provided written informed consent to use their biopsy.

### Cell lines and cell culture

Human CRC cell lines (LoVo, HCT116, RKO, Caco2, HT-29, LS-174-T, SW480 and SW620) and mouse CRC cell lines (MC38 and CT26) were obtained from the American Type Culture Collection (ATCC). RPMI1640 regular medium (Gibco, USA) supplemented with 10% regular foetal bovine serum (FBS, Gibco, USA) and antibiotics (Gibco, USA) was used to culture cells.^27^ TNF-α was configured to a final concentration of 50 ng/ml^28^ using autoclaved ddH_2_O containing 1‰ Bovine Serum Albumin (BSA, Sigma–Aldrich, USA) to treat CRC cells for inducing epithelial-mesenchymal transition (EMT).

### Western blotting

Total protein was isolated using RIPA buffer (Amresco, USA) containing protease inhibitor cocktail. Protein extract was separated on SDS-PAGE gels (Amresco, USA) followed by transfer to polyvinylidene fluoride membranes. The membranes were subsequently blocked in 5% defatted milk and incubated with primary antibody overnight at 4 °C. Following incubation with the appropriate secondary antibody conjugated to horseradish peroxidase, the blots were visualised using the enhanced chemiluminescence (FDbio-pico ECL, China). The following antibodies were included in western blotting: anti-S100P (Abcam #ab133554, UK), anti-SLC2A5 (Abcam #ab36057, UK), anti-E-cadherin (Cell Signaling Technology #3195, USA), anti-N-cadherin (Cell Signaling Technology #13116, USA), anti-Vimentin (Cell Signaling Technology, #5741, USA), anti-GAPDH (Proteintech #10494-1-AP, USA), anti- Mouse IgG (Cell Signaling Technology #5873, USA) and anti-Rabbit IgG (Cell Signaling Technology #14708, USA).

### Quantitative real-time polymerase chain reaction (qRT-PCR)

Total RNAs were extracted with TRIzol reagent (TaKaRa, Japan) according to the manufacturer’s instructions. cDNAs were synthesised with PrimeScript RT-PCR Kit (TaKaRa, Japan). Quantitative PCR with SYBR^TM^ Premix Ex Taq II Kit (TaKaRa, Japan) on the LightCycler 96 Detection System (Roche) using GAPDH for normalisation. The primers sequences used for qRT-PCR were listed in Supplementary Table 1.

### Stable cell lines construction and plasmids transfection

Lentiviral vectors plasmids were constructed by GENECHEM Biotech at Shanghai, China (http://genechem.bioon.com.cn/) or GenePharma at Shanghai, China (http://www.genepharma.com/). The GV248-Vector, GV248-SLC2A5-shRNA, CV186-Vector, CV186-SLC2A5, GV358-Vector and GV358-S100P were purchased from GENECHEM Biotech. The pGLV3-H1-GFP-Puro-Vector and pGLV3-H1-GFP-Puro-S100P-shRNA were obtained from GenePharma. The GV248-Vector, CV186-Vector, GV358-Vector and pGLV3-H1-GFP-Puro-Vector plasmids were used as the control, respectively. The shRNA sequences for SLC2A5 and S100P are shown in Supplementary Table 1.

For the packaging and harvesting procedures of lentiviruses, briefly, linearised vectors were obtained by digestion with restriction enzymes. Then the linearised vectors and the target gene fragments were circularised in vitro to generate recombinant plasmids. After identification by sequencing, the vector plasmids carrying the target gene and the packaging vectors were co-transfected into 293 T cells. After 48 h, the recombinant lentiviruses were obtained after harvesting and concentrating. To generate stably transfected cell lines, cells seeded in 24-well plates were transduced with lentiviruses for 24 h, then cells were selected with puromycin (4 μg/ml) 48 h after transduction for 7 days and expanded. The infection efficiency was validated by western blotting and qRT-PCR.

For transient transfection, Lipofectamine 2000 reagent (Invitrogen, USA) was used according to the manufacturer’s instructions. Cells were harvested post transfection with SLC2A5 luciferase plasmids (10 μg) or empty vector (10 μg) for 48 h.

### Immunohistochemistry (IHC)

IHC staining of paraffin-embedded human CRC tissue or mice liver tissue sections were performed according to standard protocols. Deparaffinised sections were rehydrated; the endogenous peroxidase activity was blocked; and the sections were subjected to antigen retrieval and blocking procedures. The samples were incubated with primary antibody overnight at 4 °C. Next day, the sections were incubated with secondary antibody for 1 h and visualised using a DAB kit (Maixin, China).

All stained sections were scored by two experienced pathologists. The expression levels were calculated using the following equation: IHC score = percentage of positive cells × staining intensity. At least three individual fields (40×) were randomly chosen to calculate a mean percentage of proportion value of staining-positive cells for each sample. The percentage of positive cells was defined as follows: 1, <10%; 2, 10–35%; 3, 35–70%; 4, å 70%. The staining intensity was evaluated as follows: 0, no staining; 1, weak staining; 2, moderate staining; 3, strong staining. When the expression score was higher than the average score, SLC2A5 or S100P expression was defined as positive, otherwise, it was defined as negative.^29,30^

### Cell proliferation assays

For CCK8 assays, when the cells were grown to 70–80% confluence, the adherent cells were digested with trypsin and harvested. Cells (1000 per well) were cultivated on 96-well plates and cell proliferation were detected for 6 days with Cell Counting kit-8 (DOJINDO Laboratories, Japan) at 450 nm according to the manufacturer’s protocols.

For colony formation assays, cells (500 per well) were cultivated on 6-well plates and cultured for 12 days. The culture was terminated until colony formation was visible to the naked eye. Colonies formed were washed with phosphate buffer, fixed in methanol and stained with 0.1% crystal violet. ImageJ software was used for cell colonies counting.

### Cell migration and invasion assays

For wound healing assays, cells were cultivated on 6-well plates and incubated to near 90% confluence. Following two washes with phosphate buffer, scratch wounds were produced in each well using a 1 ml plastic pipette tip, followed by 48 h of starvation. An inverted microscope (OLYMPUS DP22, Japan) was used to capture photos of the cells migrating at the corresponding wound sites at 0 h and 48 h.

For transwell assays, detection of migration or invasion abilities of cells were performed using transwell chambers (8-μm pore, Corning, USA) pre-coated without or with Matrigel (BD Biosciences, USA). 1.0 × 10^5^ cells were seeded into the 8-μm pore upper chambers in serum-free RPMI1640 and incubated in RPMI1640 with 10% FBS of the lower chamber of 24-well plates. Following 24 h (migration assay) or 48 h (invasion assay) of incubation, cells on the upper surface of the membrane filter were fixed with methanol and then stained with hematoxylin.^31^ Images were taken with OLYMPUS DP22 microscope and cells were quantified under at least five random microscopic fields.

### Immunofluorescence (IF)

Cells were cultured in the confocal dish for 48 h and then washed with phosphate buffer for three times and fixed in 4% paraformaldehyde for 30 min at room temperature, permeabilised with 0.3% Triton X-100 for 10 min, followed by blocking with 5% goat serum blocking solution for 30 min. anti-E-cadherin (Cell Signaling Technology #3195, USA), anti-N-cadherin (Cell Signaling Technology #13116, USA), anti-S100P (Abcam #ab133554, USA), Alex Fluor®488 goat anti-rabbit IgG and Fluor®594 goat anti-rabbit IgG (Millipore, USA) were used as primary and secondary antibodies, respectively. Nuclei were counterstained with DAPI (4, 6-diamidino-2-phenylindole), and cells were observed under a confocal laser-scanning microscope (Carl Zeiss, Germany) and photographed.

### Chromatin immunoprecipitation (ChIP)

ChIP assays were performed using Chromatin Immunoprecipitation kit (Chromatin, USA) according to the manufacturer’s protocols. Immunoprecipitation reactions were performed with 5 μg antibody against S100P (OriGene #UM500024, USA) or with IgG, used as a negative control. Purified DNA was suspended subsequently for following qRT-PCR analysis using primers of SLC2A5 promoter. Five pairs of primers for the SLC2A5 promoter for ChIP-qPCR analysis were designed by Primer 5.0 software and listed in Supplementary Table 1.

### Luciferase reporter assay

The target genes were obtained by digestion with KpnI/XhoI enzyme. Wild-type and mutant-type reporter plasmids of SLC2A5 promoter were constructed and cloned into the GV238-Vector (GENECHEM Biotech, China). Primer sequences for identification of recombinant clones were listed in Supplementary Table 1. The reporter plasmids or empty vector were transfected into SW480 and Caco2 cells using Lipofectamine 2000. After 48 h of transfection, the cells were lysed, and the fluorescence intensity of firefly or renilla fluorescein were detected using Dual-Luciferase report system (Promega, USA) according to the manufacturer’s instructions. Finally, standardised analysis of enzyme activity was performed.

### Methylation specific PCR (MSP) and bisulphite sequencing PCR (BSP)

Primers of MSP and BSP were designed using the online MethPrimer software (http://www.urogene.org/). Related primer sequences were listed in Supplementary Table 1. For MSP analysis, EpiTect Bisulphte kit (Qiagen, Germany) was applied to conduct the bisulphite modification of DNA according to the manufacturer’s protocols. The purified DNA was collected for PCR amplification, and the PCR products were electrophoresed on 2.5% agarose gels and developed with ethidium bromide.

For BSP analysis, 1 μg of genomic DNA was converted using the ZYMO EZ DNA Methylation-Gold kit (Zymo Research, USA) and one twentieth of the elution products were used as templates for PCR amplification with 35 cycles using KAPA 2 G Robust HotStart PCR Kit (Kapa Biosystems, USA). For each sample, BSP products of multiple genes were pooled equally, 5’-phosphorylated, 3’-dA-tailed and ligated to barcoded adapter using T4 DNA ligase (NEB, USA). Barcoded libraries from all samples were sequenced on Illumina platform.

### Animal model

Male C57BL/6 mice (4 weeks old) and male BALB/c mice (4 weeks old), purchased from the Animal Center of Guangdong Province, were used to construct an intrasplenic inoculation model to observe liver metastasis. All animals care and experiments were approved by the Institutional Animal Care and Use Committee (IACUC) of Nanfang Hospital. All animal studies were complied with relevant ethical regulations for animal testing and research. Six mice per group were randomly housed in 375 × 160 × 180 mm cages (FENGSHI, Suzhou, China) and given 5 days to adapt to the housing conditions. The animals were fed an autoclaved laboratory rodent diet. The environmental conditions were a temperature of 23 ± 2 °C and a humidity of 50 ± 10%.

For intrasplenic inoculation, each mouse of the experimental or the control group was subjected to the following procedures. First, the mouse was anesthetised by intraperitoneal injection using 0.1% pentobarbital sodium (Huabo Deyi Biotechnology Co., Ltd, Beijing, China) at a dose of 5 mg/100 g BW. After observing the anaesthesia reaction in mouse and confirming the success of anaesthesia, the skin was disinfected with medical alcohol. A small animal surgical instrument (PuLun Medical Devices Co., Ltd., Shanghai, China) was then used to make a small incision in the left abdomen of mouse to find the spleen. After finding the spleen, tumour cells were injected using 1-ml medical syringe (MAISINUO, Haikou, China) into the lower pole of the spleen. SLC2A5-overexpressed (LV-SLC2A5) or control (LV-NC) MC38 cells were injected at a final concentration of 1 × 10^6^ cells/50 μl PBS into spleens of C57BL/6 mice, respectively. And SLC2A5-overexpressed (LV-SLC2A5) or control (LV-NC) CT26 cells were injected at a final concentration of 1 × 10^6^ cells/50 μl PBS into spleens of BALB/c mice, respectively. Medical cotton balls were used to compress the injection site to stop bleeding. After no obvious bleeding was observed, the muscle and skin were sewed in turn. Animals were returned to the home cage until death. According to the AVMA Guidelines for the Euthanasia of Animals, animals were killed by cervical dislocation method in the laboratory after 4 weeks. The spleens and livers were dissected, and the numbers of metastatic nodules on the liver surfaces were recorded. Then the tissues were fixed in 10% neutral-buffered formalin for following haematoxylin and eosin staining (H&E) staining and IHC to confirm the pathological features and SLC2A5 expression.^27,32^

### Statistical analysis

Statistical analysis was performed using the SPSS 13.0 software (SPSS Inc, USA). *P* value < 0.05 was considered significant. All results were shown as mean ± SEM. Two-tailed, unpaired or paired Student’s *t*-test was used to compare the variables of two groups. One-way or two-way ANOVA was performed for multi-group comparisons. Linear regression analysis was performed to assess the correlation between S100P and SLC2A5.

## Results

### SLC2A5 is upregulated in CRC tissues

**Fig. 1:**
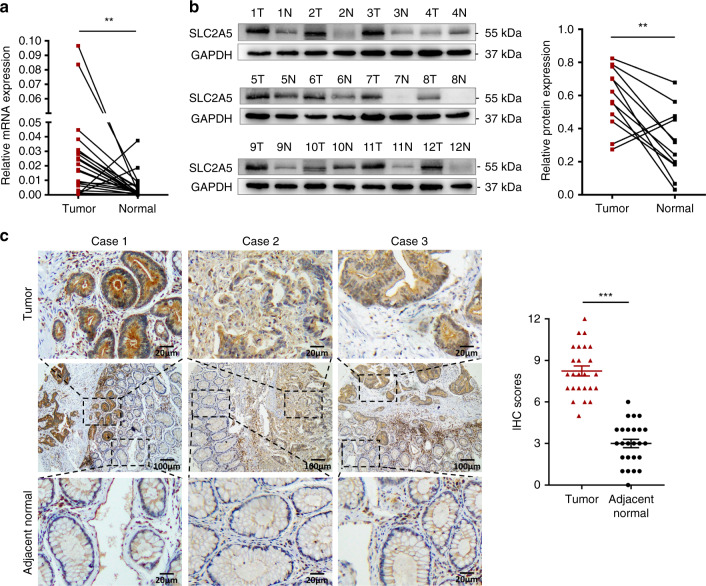
SLC2A5 is upregulated in CRC tissues. **a** qRT-PCR analysis of SLC2A5 mRNA expression in paired CRC tissues. Results are shown as mean ± SEM (*n* = 24). **P < 0.01, based on paired Student’s *t*-test. **b** Western blotting of SLC2A5 protein expression in 12 pairs of CRC tissues, GAPDH was loaded as a control. ImageJ software was used to analyse the grey values of bands to calculate relative protein expression. *P < 0.05, based on paired Student’s *t-*test. **c** IHC staining of SLC2A5 on tumour and adjacent normal tissues (inserts show ×4 magnification). Scale bar, 100 μm (10×), 20 μm (40×). Results are shown as mean ± SEM (*n* = 25). ***P < 0.001, based on paired Student’s *t*-test.

Based on the results of ChIP-sequencing analysis of nuclear S100P, as previously described, we identified the genes recruited by S100P and found that S100P interacted most strongly with SLC2A5 promoter (Supplementary Table 2). To explore the expression of SLC2A5 in CRC patients, we performed qRT-PCR and western blotting analysis and found that mRNA and protein levels of SLC2A5 were significantly increased in tumours compared to paired normal tissues (Fig. 1a, b). To further validate the protein expression of SLC2A5 in CRC patients, we performed IHC analysis on 25 pairs of tumour and adjacent normal tissues. The results showed that strong SLC2A5 signal was found in almost 75% tumour tissues, while almost all adjacent normal tissues showed weak or absent SLC2A5 expression. In addition, we also found that in the normal intestinal mucosal gland cells, SLC2A5 was mainly expressed on the cell membrane, while in the tumour cells, SLC2A5 was expressed both on the cell membrane and in the cytoplasm. Interestingly, we also observed strong staining of SLC2A5 in stromal cells (Fig. 1c). Collectively, these findings suggested that SLC2A5 expression is upregulated in colorectal tumour tissues.

### SLC2A5 promotes CRC cells invasion and migration in vitro

**Fig. 2:**
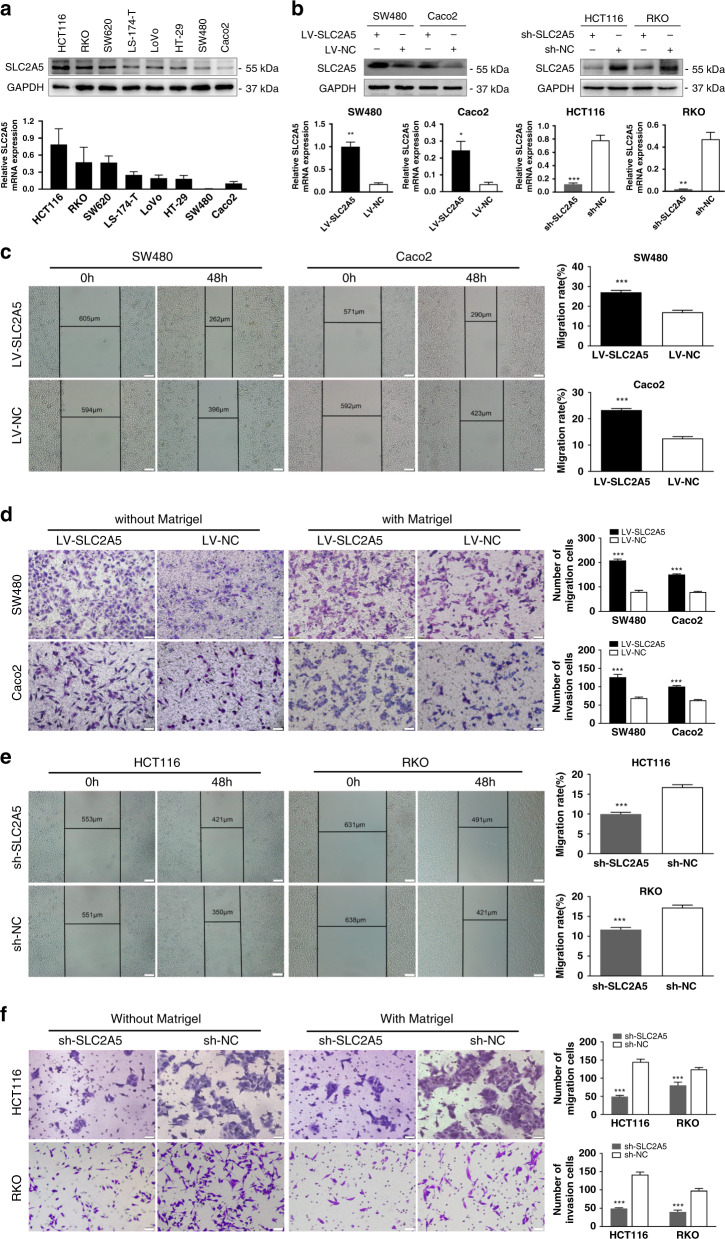
SLC2A5 promotes CRC cells invasion and migration in vitro. **a** SLC2A5 protein and mRNA expression of CRC cell lines were measured by western blotting (upper) and qRT-PCR (lower). Results are shown as mean ± SEM (*n* = 3). **b** Transfection efficiency was detected by western blotting (upper) and qRT-PCR (lower). Results are shown as mean ± SEM (*n* = 3). *P < 0.05, **P < 0.01, ***P < 0.001, based on Student’s *t*-test. **c** In SW480 and Caco2 cells that stably overexpressed SLC2A5 (LV-SLC2A5) or control vector (LV-NC), migration ability was measured by wound healing assays. Scale bar, 100 μm (10×). Results are presented as mean ± SEM (*n* = 3). ***P < 0.001, based on Student’s *t*-test. **d** In SW480 and Caco2 cells that stably expressed LV-SLC2A5 or LV-NC, migration and invasion abilities were measured by transwell assays. Scale bar, 50 μm (20×). Results are presented as mean ± SEM (*n* = 3). ***P < 0.001, based on Student’s *t*-test. **e** In HCT116 and RKO cells that stably knocked down SLC2A5 (sh-SLC2A5) or control vector (sh-NC), migration ability was measured by wound healing assays. Scale bar, 100 μm (10×). Results are presented as mean ± SEM (*n* = 3). ***P < 0.001, based on Student’s *t*-test. **f** In HCT116 and RKO cells that stably expressed sh-SLC2A5 or sh-NC, migration and invasion abilities were measured by transwell assays. Scale bar, 50 μm (20×). Results are presented as mean ± SEM (*n* = 3). ***P < 0.001, based on Student’s *t*-test.

Given previous results, we further assessed the functional roles of SLC2A5 in CRC cells. We first detected protein and mRNA expression of SLC2A5 in various CRC cell lines by western blotting and qRT-PCR (Fig. 2a). SLC2A5-expressing recombinant lentivirus (LV-SLC2A5) and vector (LV-NC) were used to establish SLC2A5-overexpressed SW480 and Caco2 cells, while SLC2A5-targeting shRNA (sh-SLC2A5) or corresponding vector (sh-NC) was introduced into HCT116 and RKO cells with relatively high endogenous SLC2A5 expression (Fig. 2b). Results of CCK8 and colony formation assays showed that the different expression levels of SLC2A5 had no significant effects on cell proliferation (Supplementary Fig. S1). Next, we investigated the effects of SLC2A5 on cell migration and invasion using wound healing assays and transwell assays. The results showed that the ectopic expression of SLC2A5 significantly increased the migration and invasion of CRC cells (Fig. 2c, d). In contrast, knockdown of SLC2A5 significantly suppressed the migration and invasion capabilities of CRC cells (Fig. 2e, f). Collectively, these results suggested that SLC2A5 promotes CRC cells invasion and migration in vitro.

### SLC2A5 promotes CRC cells metastasis in vivo

**Fig. 3:**
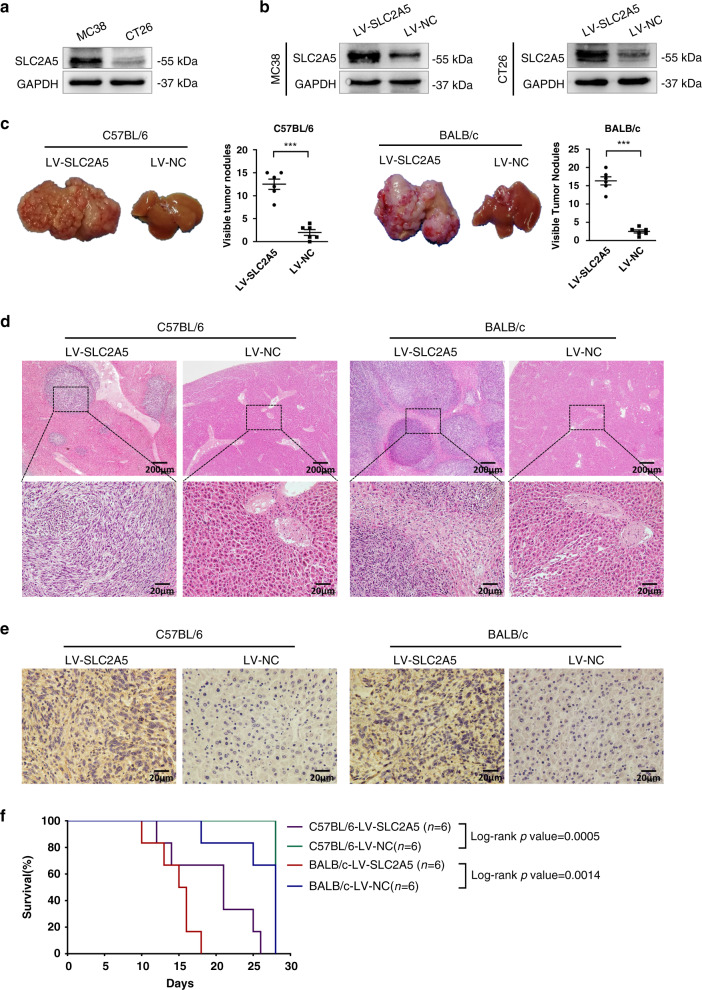
SLC2A5 promotes CRC cells metastasis in vivo. **a** The endogenous expression levels of SLC2A5 in MC38 and CT26 cells were detected by western blotting. **b** Protein expression levels of SLC2A5 in MC38 and CT26 cells that expressed LV-SLC2A5 or LV-NC were detected using western blotting. **c** Intrasplenic injection model was used to evaluate the in vivo effects of SLC2A5 on tumour metastasis. Representative images of livers derived from C57BL/6 and BALB/c mice injected with LV-SLC2A5 or LV-NC transfected MC38 or CT26 cells are shown. Formation of metastatic nodules at the liver surfaces are summarised in the right panels. Results are presented as mean ± SEM (*n* = 6). ***P < 0.001, based on Student’s *t*-test. **d** Representative images of H&E-stained sections derived from the liver metastatic nodules. The sections of liver derived from mice that injected with LV-NC cells were used as control. Scale bar, 200 μm (4×), 20 μm (40×). **e** Representative IHC images of SLC2A5 expression in the liver sections. Scale bar, 20 μm (40×). **f** Survival curves of mice in different groups.

To evaluate the in vivo effects of SLC2A5 on tumour metastasis, we constructed intrasplenic inoculation model in mice. We first detected the endogenous expression of murine MC38 and CT26 cells by western blotting and then constructed MC38-LV-SLC2A5 and MC38-LV-NC cells, as well as CT26-LV-SLC2A5 and CT26-LV-NC cells (Fig. 3a, b). MC38-LV-SLC2A5 or MC38-LV-NC cells were injected into spleens of C57BL/6 mice, and CT26-LV-SLC2A5 or CT26-LV-NC cells were injected into spleens of BALB/c mice, with six mice each group. Continuous observation for 4 weeks and recording the survival time of mice. After 4 weeks the mice were sacrificed and the metastatic nodules at the liver surfaces were counted. We found a significantly larger number of metastatic nodules were induced at the surface of the livers of mice injected with the SLC2A5-overexpressed cells than those with the cells of control group (Fig. 3c). H&E staining confirmed that the nodules on the surfaces of mice livers were metastatic tumours (Fig. 3d). Results from the IHC staining confirmed the expression of SLC2A5 in liver metastatic lesions that developed from the SLC2A5-transfected cells (Fig. 3e). The survival curves indicated that the overall survival time of mice in LV-SLC2A5 group was shorter than that of LV-NC group (Fig. 3f). Taken together, these results supported the view that SLC2A5 promotes CRC cells metastasis in vivo.

### SLC2A5 facilitates EMT in CRC cells

EMT has been reported to allow cells to acquire migratory and invasive behaviours during many biological processes, such as embryonic development, fibrosis and cancer metastasis.^33^ Therefore, we would like to further explore whether the high expression of SLC2A5 is related to EMT in CRC cells. We performed western blotting to detected the protein expression levels of EMT-related markers in CRC cells with different SLC2A5 expression. In SLC2A5-overexpressed SW480 and Caco2 cells, E-cadherin was downregulated, while N-cadherin and Vimentin were upregulated. The expression levels of these markers were reversed by the knockdown of SLC2A5 in HCT116 and RKO cells (Supplementary Fig. S2). Notably, when SLC2A5 was overexpressed in SW480 and Caco2 cells, we found that the morphology of some cells transitioned from an epithelial-like form to a spindle-shaped or elongated, mesenchymal form, which indicated that SLC2A5 might function to promote the transformation of SW480 and Caco2 cells from epithelial status to mesenchymal status (Supplementary Fig. S3a). IF analysis showed that SW480 and Caco2 cells with SLC2A5 overexpression displayed reduced epithelial marker E-cadherin and increased mesenchymal marker N-cadherin. In contrast, the epithelial marker was upregulated, and the mesenchymal marker was downregulated in HCT116 and RKO cells with SLC2A5 knockdown (Supplementary Fig. S3b). Therefore, we speculated that EMT might be involved in the promotion effect of SLC2A5 on CRC cell invasion and migration. To evidence that EMT is essential for SLC2A5-mediated CRC cell migration, we treated cells with TNF-α (50 ng/ml) to induce EMT. We cultured SW480-LV-SLC2A5, SW480-LV-NC, Caco2-LV-SLC2A5 and Caoc2-LV-NC cells in presence of TNF-α, and detected the protein levels of E-cadherin, N-cadherin and Vimentin. The results showed that the expression of E-cadherin was downregulated, while N-cadherin and vimentin were upregulated in LV-NC cells treated with TNF-α. LV-SLC2A5 cells without TNF-α treatment also showed the same changes. Furthermore, LV-SLC2A5 cells treated with TNF-α had the most significant downregulation of E-cadherin, and upregulation of N-cadherin and Vimentin (Supplementary Fig. S4a). Results of migration assays showed that the migration ability of LV-NC cells treated with TNF-α and LV-SLC2A5 cells without TNF-α treatment was similarly enhanced, while LV-SLC2A5 cells induced by TNF-α had the most obvious migration (Supplementary Fig. S4b). Taken together, these results indicated that SLC2A5 could promote CRC cell migration by inducing EMT.

### SLC2A5 is a downstream functional target of S100P to promote cell invasion and migration

**Fig. 4:**
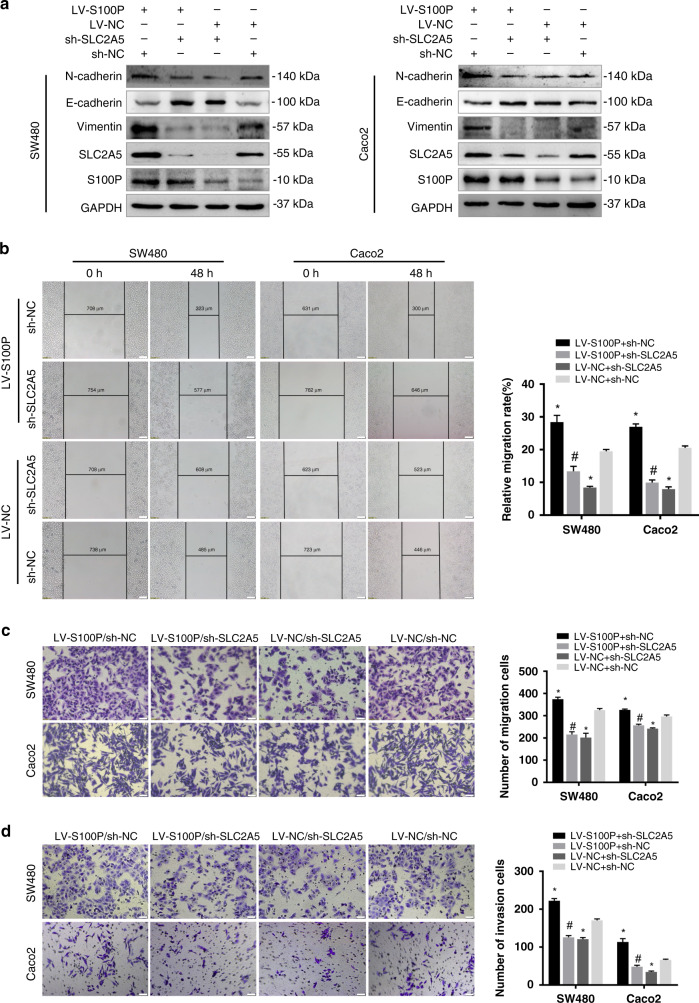
Knockdown of SLC2A5 in S100P-overexpressed cells suppresses invasion and migration. SLC2A5 shRNA (sh-SLC2A5) or vector (sh-NC) was transfected in SW480 and Caco2 cells that stably overexpressed S100P (LV-S100P) or vector (LV-NC). **a** Protein expression levels of N-cadherin, E-cadherin, Vimentin, SLC2A5, S100P and GAPDH were detected using western blotting. **b–d** The migration and invasion abilities were measured using wound healing assays (10×) (**b**) and transwell assays (20×) without (**c**) or with (**d**) Matrigel. Scale bar, 100 μm (10×), 50 μm (20×). Results are shown as mean ± SEM (*n* = 3). *P < 0.01, compared to LV-NC + sh-NC; ^#^*P* < 0.01, compared to LV-S100P + sh-NC. Student’s *t*-test were used to analyse the data.

To determine whether SLC2A5 expression is regulated by S100P, we detected the expression of SLC2A5 in CRC cell lines with different expression levels of S100P using western blotting and qRT-PCR. The results showed that the SLC2A5 expression was significantly upregulated in S100P-overexpressed cells, and the expression of SLC2A5 was suppressed when S100P was knocked down (Supplementary Fig. S5a, b). In our previous study, we found that S100P could promote CRC cells invasion and metastasis.^13^ Here, we would like to further explore whether SLC2A5 is a downstream functional target of S100P. To confirm our view, we knocked down SLC2A5 in SW480 and Caoc2 cells that stably overexpressing S100P. Results of western blotting showed that the expression of E-cadherin suppressed by the overexpression of S100P was substantially increased, while N-cadherin and Vimentin were reduced following transfected with SLC2A5 shRNA (Fig. 4a). In addition, transfection with SLC2A5 shRNA significantly inhibited the promoting effect of S100P on CRC cell migration and invasion (Fig. 4b–d). We also overexpressed SLC2A5 in HCT116 and RKO cells with stable knockdown of S100P. Results of western blotting showed that after overexpression of SLC2A5, the expression of E-cadherin, which was upregulated due to S100P knockdown, was significantly reduced, while the expression of N-cadherin and Vimentin was upregulated (Supplementary Fig. S6a). Furthermore, the overexpression of SLC2A5 significantly restored the inhibitory effect of S100P-shRNA on the migration and invasion of CRC cells (Supplementary Fig. S6b, c, d). These findings suggested that SLC2A5 is a functional target for S100P to promote CRC cell invasion and migration.

### S100P integrates to the SLC2A5 promoter and reduces its methylation levels to enhance SLC2A5 transcription

**Fig. 5:**
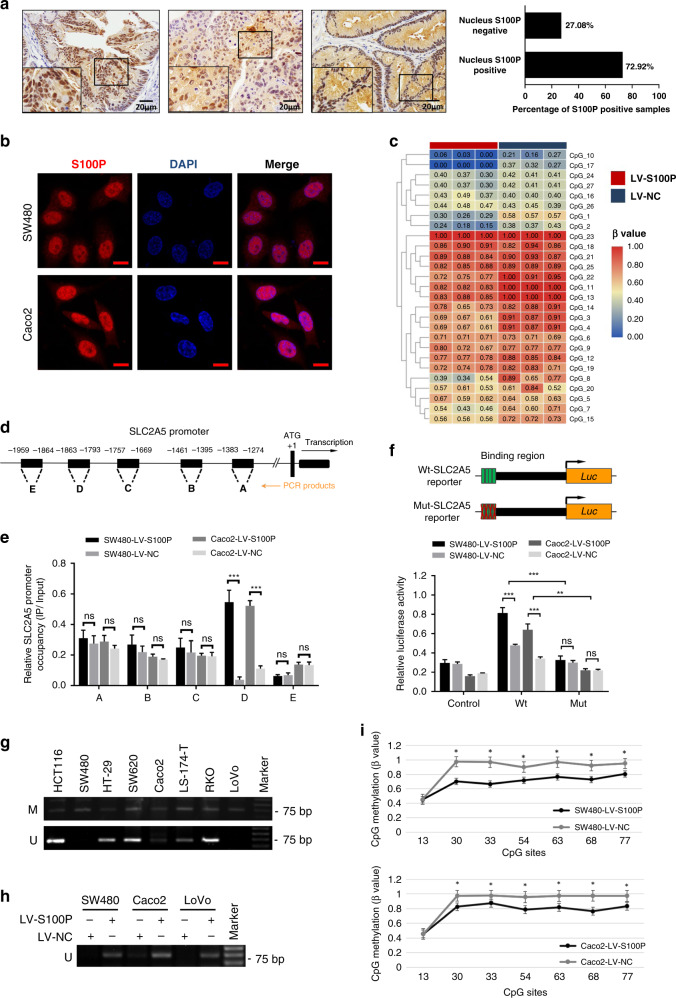
S100P integrates to SLC2A5 promoter and reduces its methylation levels to promote SLC2A5 transcription. **a** Representative IHC images of tissue microarray (TMA), showing the positive expression of S100P in the nucleus. Scale bar, 20 μm (40×). The histogram shows the proportion of nuclear S100P-positive or nuclear S100P negative samples in S100P-positive CRC tissues. **b** IF analysis of subcellular localisation of S100P in SW480 and Caco2 cells. Scale bar, 10 μm (100×). **c** Heatmap shows methylation mass spectrometry sequencing of the SLC2A5 promoter in SW480-LV-S100P or SW480-LV-NC cells. **d** Schematic representation of the SLC2A5 promoter, showing the translational start site. The five regions used for chromatin immunoprecipitation (ChIP) are also indicated. **e** ChIP analysis showing the occupancy of S100P on the SLC2A5 promoter in SW480 or Caco2 cells with S100P overexpression or control vector. Data are shown as fold enrichment relative to input and mean ± SEM (*n* = 3). ***P < 0.001, based on Student’s *t*-test. **f** Schematic diagram showing the reporters of wild-type (Wt) or mutant (Mut) SLC2A5 promoter. The binding region of S100P on the SLC2A5 promoter was mutated (upper). The reporter gene system was used to detect the luciferase activity of SW480 and Caoc2 cells with LV-S100P or LV-NC (lower). Results are shown as mean ± SEM (*n* = 3). **P < 0.01, ***P < 0.001, based on Student’s *t*-test or two-way ANOVA. **g** MSP analysis was used to detect the methylation status of S100P binding region on the SLC2A5 promoter in indicated CRC cell lines. M methylated, U unmethylated. **h** MSP was used to detect the methylation status of S100P binding region on the SLC2A5 promoter in SW480, Caco2 and LoVo cells with LV-S100P or LV-NC. **i** BSP analysis of methylation levels at CpG sites of S100P binding region on the SLC2A5 promoter in SW480, Caco2 cells with LV-S100P or LV-NC. Results are shown as mean ± SEM (*n* = 3). ***P < 0.001, based on Student’s *t*-test.

Next, we investigated the regulatory mechanism of SLC2A5 expression mediated by S100P. We confirmed the positive expression of S100P in the nucleus of CRC tissues by IHC staining (Fig. 5a). Consistently, IF analysis of the subcellular localisation of S100P showed that the S100P signal was apparently concentrated in the nucleus of SW480 and Caco2 cells (Fig. 5b). To determine whether the methylation levels of SLC2A5 promoter were affected by the expression of S100P, we performed methylation mass spectrometry sequencing analysis on the SLC2A5 promoter in SW480-LV-SLC2A5 or SW480-LV-NC cells, results revealed that the ectopic S100P expression significantly reduced the methylation levels of SLC2A5 promoter (Fig. 5c). To identify the certain position where S100P integrates to SLC2A5 promoter, we constructed the corresponding primers for differentially methylated regions of SLC2A5 promoter based on the result of methylation mass spectrometry (Fig. 5d). ChIP-qPCR analysis showed that in the sequence ranging from −1863 to −1793bp on the SLC2A5 promoter, the relative enrichment (IP/input) of SW480-LV-SLC2A5 cells was significantly increased compared to SW480-LV-NC cells (Fig. 5e). Based on these results, we constructed the wild-type and mutant SLC2A5 promoter reporter plasmids and analysed the effect of S100P on the transcription of SLC2A5. Luciferase reporter gene system showed that the luciferase activity of the wild-type SLC2A5 promoter was significantly stronger than that of the mutant SLC2A5 promoter, and ectopic S100P expression further enhanced the transcription of SLC2A5 (Fig. 5f). Next, we synthesised methylation and unmethylation primers and performed MSP analysis to detect the methylation status of S100P specific binding region on the SLC2A5 promoter in different CRC cell lines. The results showed that all cells except SW480, Caco2 and LoVo showed the demethylation status (Fig. 5g), which were associated with relatively high endogenous expression of SLC2A5 (Fig. 2a). In order to confirm the demethylation effect of S100P on the binding region of SLC2A5 promoter, we performed S100P overexpression in SW480, Caco2 and LoVo cells. MSP analysis revealed that the ectopic S100P expression transformed the methylation status of specific binding region on the SLC2A5 promoter to demethylation (Fig. 5h). BSP analysis showed that among seven CpG sites of S100P specific binding region on the SLC2A5 promoter, the methylation levels of six CpG sites were significantly reduced due to the ectopic S100P expression (Fig. 5i). Taken together, these results indicated that the transcriptional activity of SLC2A5 is regulated by S100P-dependent SLC2A5 promoter demethylation.

### Validation of the correlation between S100P and SLC2A5 in clinical CRC samples

**Fig. 6:**
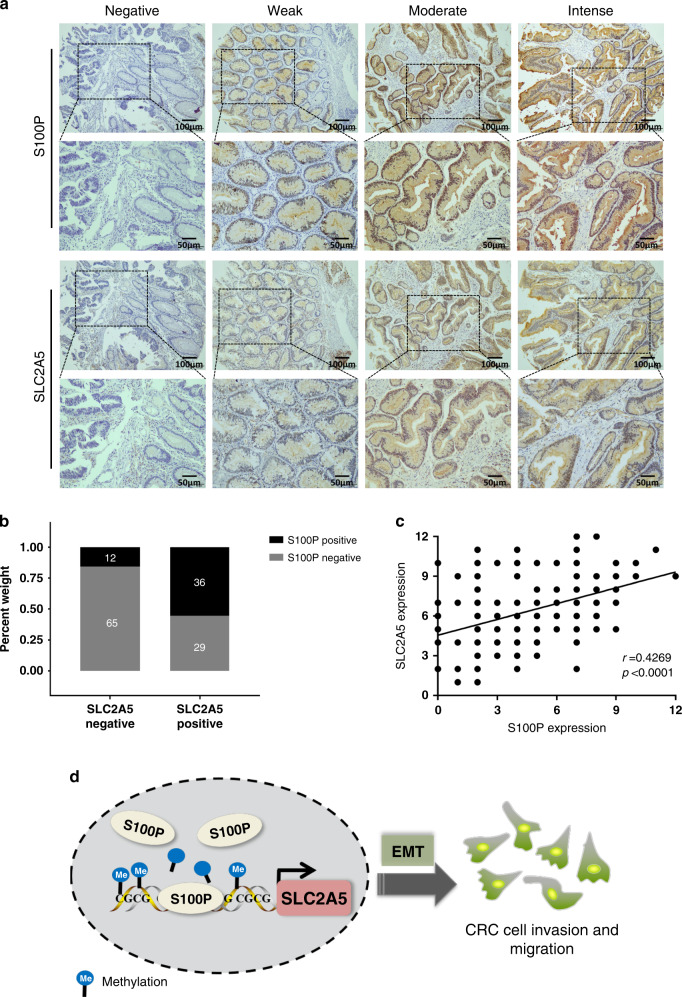
Validation of the correlation between S100P and SLC2A5 in clinical CRC samples. **a** Representative IHC images of S100P and SLC2A5 expression in TMA. Scale bar, 100 μm (10×), 50 μm (20×). **b** The stacked histogram shows the expression distribution of S100P and SLC2A5 in 142 patients with CRC. **c** Correlation analysis shows a positive correlation between S100P and SLC2A5 expression in primary CRC (*n* = 142), based on Pearson χ^2^ test. **d** Schematic diagram summarising the mechanism in this study, namely, nuclear S100P reduces the methylation levels of SLC2A5 promoter by binding to the SLC2A5 promoter, thereby enhancing the transcriptional activity of SLC2A5, followed by promoting CRC cell invasion and metastasis via inducing EMT.

We detected the expression of S100P and SLC2A5 in TMA composed of 142 human CRC samples by IHC, and evaluated the correlation between the expression of S100P and SLC2A5 (Fig. 6a). The results showed that in the SLC2A5 negative expression (negative and weak staining) group, 84.4% (65/77) of the patients also showed negative expression of S100P, while in the patients with positive SLC2A5 expression (moderate and intense staining), 55.4% (36/65) of patients showed positive expression of S100P (Fig. 6b). Spearman’s correlation analysis revealed a positive correlation between S100P and SLC2A5 expression (*r* = 0.4269, *P* < 0.0001) (Fig. 6c). These results suggested that there is a certain correlation between the expression of S100P and SLC2A5 in CRC patients.

## Discussion

SLC2A belongs to the solute carrier 2 family with 14 isomers (SLC2A1–14) have been currently identified, which are involved in transmembrane transport of carbohydrates.^34^ SLC2A expression was significantly upregulated in tumour cells and promoted glucose metabolism.^35^ According to the location of extracellular long loops in the structure of SLC2A proteins, SLC2A can be divided into three types. SLC2A5 belongs to the second class of transporters, which extracellular long-loops locate on transmembrane domains 2.^36^ Initially, studies reported that SLC2A5 was related to the occurrence of diabetes.^37^ In recent years, the roles of SLC2A5 in tumour progression have received increasing attention. As we mentioned before, previous studies consistently approved that the expression of SLC2A5 was upregulated in several types of human cancers such as breast cancer, lung cancer, renal cell carcinoma and glioma. Despite substantial evidence indicating that SLC2A5 is associated with multiple cancers, the mechanism of SLC2A5 in cancer remains elusive. It is reported that in clear cell renal cell carcinoma, high expression of SLC2A5 increased tumour cells growth, while the deletion of SLC2A5 dramatically attenuated cellular malignancy via activating the apoptotic pathway.^38^ Some articles claimed that SLC2A5-mediated fructose utilisation drove lung cancer growth by stimulating fatty acid synthesis and AMPK/mTORC1 signaling.^39^ Therefore, it seemed that the studies involving the mechanism of SLC2A5 were still limited to the downstream level of the molecule. In CRC, only one study more than two decades ago reported that treatment of differentiated Caco2 cells with forskolin could stimulate adenylate cyclase and raise intracellular cyclic AMP levels to raise SLC2A5 expression by *cis*-acting regulatory sequences.^40^ However, this study was not widely persuasive due to the limitations of cell line selection. As the roles of SLC2A5 in CRC remain obscure, we hence attempted to conduct a more comprehensive study of expression, functional roles and regulatory mechanism of SLC2A5 in CRC.

In the present study, we found that SLC2A5 was generally upregulated in human CRC. Furthermore, on functional verification, our gain-of-function and loss-of-function experiments in vitro and in vivo clearly indicated the metastasis-promoting role of SLC2A5 in CRC, although SLC2A5 had no effect on the proliferation of CRC cells under general culture condition.

EMT is commonly considered to be a crucial process for epithelial tumour cells to dissociate and spread to distant locations. It is characterised by the downregulation of genes encoding for epithelial cell junction proteins (E-cadherin, claudins, etc.) and the activation of some genes, among which the protein products (N-cadherin and vimentin, etc.) promote mesenchymal adhesion.^41^ The transition from epithelial to mesenchymal morphology and the loss of cell adhesion are orchestrated by various transcription factor families (EMT-TFs) such as Snail, Twist and Zeb.^42,43^ We supposed that EMT might be involved in the process of SLC2A5 regulating the distant metastasis of CRC cells. Our results indicated that SLC2A5 could directly or indirectly regulate the expression of EMT-related molecular markers. However, the underlying mechanism of SLC2A5 regulating EMT is still unclear. Glycolysis, oxidative phosphorylation, glutamine and lipid metabolism are the main energy producing pathways that maintain cellular harmony. It is reported that their metabolic disorders and reprogramming affect the initiation and progression of EMT and metastasis. Unlike normal cells, cancer cells rely more on aerobic glycolysis (also known as the Warburg effect) to meet their higher energy requirements during proliferation.^44^ Studies have reported that in some endocrine cancers, the activity of glycolysis was closely associated with the EMT phenotypes, and dysregulation of glycolytic enzymes might be an important cause of mediating EMT.^45,46^ For example, the expression of phosphate glucose isomerase (PGI) was upregulated in breast cancer, and promoted the motility, migration, metastasis and EMT of cancer cells by inducing the expression of EMT-TFs.^47^ For another example, fructose 1,6-bisphosphatase (FBP1), which catalyses the hydrolysis of fructose 1,6-bisphosphate to fructose 6-phosphate, has been shown to be a direct target of Snail and Zeb1 transcriptional repression that promoted an increase for invasiveness of cancer cells.^48,49^ In addition, some studies have reported that the expression of glucose transporters SLC2A1 and SLC2A3 was associated with increased glucose uptake, activation of EMT-TFs and tumour cell invasiveness.^50,51^ SLC2A5 is a fructose-specific transporter, which plays an essential role in the process of fructose metabolism. Studies have found that in a glucose-deficient tumour environment, acute myeloid leukaemia tumour cells could upregulate the expression of SLC2A5 to ingest a large amount of fructose to maintain a high level of glycolysis.^23^ Aldolase is an indispensable enzyme for cells to utilise fructose for glycolysis, and it has also been reported to be related to cancer cell metastasis and EMT.^52^ Therefore, we suspect that SLC2A5 may drive glycolysis and metabolic reprogramming through fructose utilisation in colorectal cancer, and then regulate EMT-TFs by affecting changes in certain glycolytic enzymes, thereby mediating EMT. However, we still need a lot of evidence to clarify our views. Our current results indicate that SLC2A5 is involved in the maintenance of the mesenchymal phenotype of CRC. Knockdown of SLC2A5 leads to upregulation of E-cadherin, downregulation of N-cadherin and Vimentin and weakening of cell invasion and migration.

S100P protein functions as extracellular and/or intracellular regulators of diverse cellular processes and participate in various human pathologies including cancer.^53^ Previous data regarding subcellular localisation of S100P differed and described either nuclear/supranuclear or cytoplasmic or membrane position depending on the cell/tissue type and experimental settings.^54–56^ Functional studies of S100P indicated that its biological activities were exerted through extracellular signalling via RAGE receptor, resulting in increased proliferation and survival,^57^ or through intracellular interaction with ezrin, leading to increased cell migration and metastasis.^58^ Studies have also pointed out that S100P binding to S100PBPR stimulated translocation of S100P to nucleus.^59^ However, the mechanism by which S100P activates or represses target genes in the nucleus remains unknown. Here, we validated the localisation of S100P in the nucleus of CRC cells and found S100P could specifically integrate to the SLC2A5 promoter region, which is rich in CpG sites. We also found that overexpressing S100P in CRC cells increased SLC2A5 expression, and the knockdown of S100P suppressed SLC2A5 expression. In addition, knockdown of SLC2A5 in S100P-overexpressed CRC cells could significantly suppress cell invasion and migration mediated by S100P, while the overexpression of SLC2A5 in S100P knockdown cells restored the invasion and migration ability of cells, indicating that SLC2A5 is a downstream functional target of S100P. Combining the above results, we hypothesised that S100P reduces the methylation levels of SLC2A5 promoter by binding to the SLC2A5 promoter, thereby promoting SLC2A5 expression.

Abnormal demethylation of DNA promoter is an important mechanism of oncogene activation. Study has reported that the demethylation of the upstream promoter of Shc3 increased the expression of Shc3 to induce EMT and promote HCC cell metastasis.^60^ Melanoma antigen (MAGE)-encoding genes were expressed in advanced gastric cancer via demethylation of promoter CpG islands.^61^ There are two main ways of DNA demethylation. The passive pathway of demethylation refers to the adhesion of protein factors in the nucleus to the DNA methylation sites, resulting in the DNA sequences near the adhesion sites not being able to interact with the methyltransferase, thereby maintaining low methylation level. Active demethylation is the process of removing methyl groups on methylated DNA by demethylase.^62^ Although several aberrantly demethylated genes related to CRC metastasis have been identified,^63^ the mechanism of SLC2A5 promoter demethylation in CRC has not been reported. Herein, through methylation mass spectrometry, MSP and BSP, we proved that the ectopic S100P expression significantly reduced the methylation levels of SLC2A5 promoter. Through ChIP-qPCR analysis, we further identified the specific binding region of S100P on the SLC2A5 promoter. Luciferase reporter gene system confirmed that S100P could promote the transcription of SLC2A5. After the specific binding region of S100P on the SLC2A5 promoter was mutated, the effect of transcriptional activation of S100P was almost completely lost. Therefore, we supposed that S100P might be involved in the promoter demethylation and transcription activation of SLC2A5. Nevertheless, our study still has some shortcomings that it fails to clarify whether S100P can promote the demethylation of SLC2A5 by recruiting demethylase to the SLC2A5 promoter.

In conclusion, we found that SLC2A5 is upregulated in CRC and its roles in promoting metastasis. Mechanistically, the transcriptional activation of SLC2A5 is regulated by nuclear S100P-mediated SLC2A5 promoter demethylation and promotes CRC cell invasion and metastasis by inducing EMT (Fig. 6d). Overall, our results provided new insights into the mechanism of tumour metastasis, and a novel theoretical basis for targeted intervention of CRC.

## Supplementary information

Supplementary Materials

## Data Availability

All data and materials generated and/or analysed during the current study are available from the corresponding author upon reasonable request.

## References

[1] Bray F, Ferlay J, Soerjomataram I, Siegel RL, Torre LA, Jemal A (2018). Global cancer statistics 2018: GLOBOCAN estimates of incidence and mortality worldwide for 36 cancers in 185 countries. CA Cancer J. Clin..

[2] Brenner H, Kloor M, Pox CP (2014). Colorectal cancer. Lancet.

[3] Deng F, Zhou R, Lin C, Yang S, Wang H, Li W (2019). Tumor-secreted dickkopf2 accelerates aerobic glycolysis and promotes angiogenesis in colorectal cancer. Theranostics.

[4] Jiao HL, Ye YP, Yang RW, Sun HY, Wang SY, Wang YX (2017). Downregulation of SAFB sustains the NF-kappaB pathway by targeting TAK1 during the progression of colorectal cancer. Clin. Cancer Res..

[5] Prica F, Radon T, Cheng Y, Crnogorac-Jurcevic T (2016). The life and works of S100P—from conception to cancer. Am. J. Cancer Res..

[6] Jiang H, Hu H, Tong X, Jiang Q, Zhu H, Zhang S (2012). Calcium-binding protein S100P and cancer: mechanisms and clinical relevance. J. Cancer Res. Clin. Oncol..

[7] Heizmann CW, Ackermann GE, Galichet A (2007). Pathologies involving the S100 proteins and RAGE. Subcell. Biochem.

[8] Koltzscher M, Neumann C, Konig S, Gerke V (2003). Ca2+-dependent binding and activation of dormant ezrin by dimeric S100P. Mol. Biol. Cell.

[9] Wu Z, Boonmars T, Nagano I, Boonjaraspinyo S, Srinontong P, Ratasuwan P (2016). Significance of S100P as a biomarker in diagnosis, prognosis and therapy of opisthorchiasis-associated cholangiocarcinoma. Int. J. Cancer.

[10] Wang X, Tian T, Li X, Zhao M, Lou Y, Qian J (2015). High expression of S100P is associated with unfavorable prognosis and tumor progression in patients with epithelial ovarian cancer. Am. J. Cancer Res..

[11] Dong L, Wang F, Yin X, Chen L, Li G, Lin F (2014). Overexpression of S100P promotes colorectal cancer metastasis and decreases chemosensitivity to 5-FU in vitro. Mol. Cell Biochem..

[12] Barry S, Chelala C, Lines K, Sunamura M, Wang A, Marelli-Berg FM (2013). S100P is a metastasis-associated gene that facilitates transendothelial migration of pancreatic cancer cells. Clin. Exp. Metastasis.

[13] Shen ZY, Fang Y, Zhen L, Zhu XJ, Chen H, Liu H (2016). Analysis of the predictive efficiency of S100P on adverse prognosis and the pathogenesis of S100P-mediated invasion and metastasis of colon adenocarcinoma. Cancer Genet..

[14] Nomura N, Verdon G, Kang HJ, Shimamura T, Nomura Y, Sonoda Y (2015). Structure and mechanism of the mammalian fructose transporter GLUT5. Nature.

[15] Thorens B, Mueckler M (2010). Glucose transporters in the 21st Century. Am. J. Physiol. Endocrinol. Metab..

[16] Burant CF, Takeda J, Brot-Laroche E, Bell GI, Davidson NO (1992). Fructose transporter in human spermatozoa and small intestine is GLUT5. J. Biol. Chem..

[17] Douard V, Ferraris RP (2013). The role of fructose transporters in diseases linked to excessive fructose intake. J. Physiol..

[18] Barone S, Fussell SL, Singh AK, Lucas F, Xu J, Kim C (2009). Slc2a5 (Glut5) is essential for the absorption of fructose in the intestine and generation of fructose-induced hypertension. J. Biol. Chem..

[19] Hirayama A, Kami K, Sugimoto M, Sugawara M, Toki N, Onozuka H (2009). Quantitative metabolome profiling of colon and stomach cancer microenvironment by capillary electrophoresis time-of-flight mass spectrometry. Cancer Res..

[20] Fan X, Liu H, Liu M, Wang Y, Qiu L, Cui Y (2017). Increased utilization of fructose has a positive effect on the development of breast cancer. PeerJ.

[21] Weng Y, Fan X, Bai Y, Wang S, Huang H, Yang H (2018). SLC2A5 promotes lung adenocarcinoma cell growth and metastasis by enhancing fructose utilization. Cell Death Discov..

[22] Su C, Li H, Gao W (2018). GLUT5 increases fructose utilization and promotes tumor progression in glioma. Biochem. Biophys. Res. Commun..

[23] Chen WL, Wang YY, Zhao A, Xia L, Xie G, Su M (2016). Enhanced fructose utilization mediated by SLC2A5 is a unique metabolic feature of acute myeloid leukemia with therapeutic potential. Cancer Cell.

[24] Lund AH, van Lohuizen M (2004). Epigenetics and cancer. Genes Dev..

[25] Portela A, Esteller M (2010). Epigenetic modifications and human disease. Nat. Biotechnol..

[26] Qu X, Sandmann T, Frierson HJ, Fu L, Fuentes E, Walter K (2016). Integrated genomic analysis of colorectal cancer progression reveals activation of EGFR through demethylation of the EREG promoter. Oncogene.

[27] Fang Y, Shen ZY, Zhan YZ, Feng XC, Chen KL, Li YS (2019). CD36 inhibits beta-catenin/c-myc-mediated glycolysis through ubiquitination of GPC4 to repress colorectal tumorigenesis. Nat. Commun..

[28] Liao SJ, Luo J, Li D, Zhou YH, Yan B, Wei JJ (2019). TGF-beta1 and TNF-alpha synergistically induce epithelial to mesenchymal transition of breast cancer cells by enhancing TAK1 activation. J. Cell Commun. Signal.

[29] Shen Z, Feng X, Fang Y, Li Y, Li Z, Zhan Y (2019). POTEE drives colorectal cancer development via regulating SPHK1/p65 signaling. Cell Death Dis..

[30] Shen Z, Li Y, Fang Y, Lin M, Feng X, Li Z (2019). SNX16 activates c-Myc signaling by inhibiting ubiquitin-mediated proteasomal degradation of eEF1A2 in colorectal cancer development. Mol. Oncol..

[31] Ren X, Yang X, Cheng B, Chen X, Zhang T, He Q (2017). HOPX hypermethylation promotes metastasis via activating SNAIL transcription in nasopharyngeal carcinoma. Nat. Commun..

[32] Tan X, Chen S, Wu J, Lin J, Pan C, Ying X (2017). PI3K/AKT-mediated upregulation of WDR5 promotes colorectal cancer metastasis by directly targeting ZNF407. Cell Death Dis..

[33] Nieto MA (2013). Epithelial plasticity: a common theme in embryonic and cancer cells. Science.

[34] Mueckler M, Thorens B (2013). The SLC2 (GLUT) family of membrane transporters. Mol. Asp. Med..

[35] Godoy A, Ulloa V, Rodriguez F, Reinicke K, Yanez AJ, Garcia ML (2006). Differential subcellular distribution of glucose transporters GLUT1-6 and GLUT9 in human cancer: ultrastructural localization of GLUT1 and GLUT5 in breast tumor tissues. J. Cell Physiol..

[36] Montel-Hagen A, Sitbon M, Taylor N (2009). Erythroid glucose transporters. Curr. Opin. Hematol..

[37] Shepherd PR, Gibbs EM, Wesslau C, Gould GW, Kahn BB (1992). Human small intestine facilitative fructose/glucose transporter (GLUT5) is also present in insulin-responsive tissues and brain. Investigation of biochemical characteristics and translocation. Diabetes.

[38] Jin X, Liang Y, Liu D, Luo Q, Cai L, Wu J (2019). An essential role for GLUT5-mediated fructose utilization in exacerbating the malignancy of clear cell renal cell carcinoma. Cell Biol. Toxicol..

[39] Chen WL, Jin X, Wang M, Liu D, Luo Q, Tian H (2020). GLUT5-mediated fructose utilization drives lung cancer growth by stimulating fatty acid synthesis and AMPK/mTORC1 signaling. JCI Insight.

[40] Mahraoui L, Takeda J, Mesonero J, Chantret I, Dussaulx E, Bell GI (1994). Regulation of expression of the human fructose transporter (GLUT5) by cyclic AMP. Biochem. J..

[41] Vu T, Datta PK (2017). Regulation of EMT in colorectal cancer: a culprit in metastasis. Cancers (Basel).

[42] Bhattacharya D, Scime A (2019). Metabolic regulation of epithelial to mesenchymal transition: implications for endocrine cancer. Front. Endocrinol. (Lausanne).

[43] Puisieux A, Brabletz T, Caramel J (2014). Oncogenic roles of EMT-inducing transcription factors. Nat. Cell Biol..

[44] Pavlova NN, Thompson CB (2016). The emerging hallmarks of cancer metabolism. Cell Metab..

[45] Daemen A, Peterson D, Sahu N, McCord R, Du X, Liu B (2015). Metabolite profiling stratifies pancreatic ductal adenocarcinomas into subtypes with distinct sensitivities to metabolic inhibitors. Proc. Natl Acad. Sci. USA.

[46] Bacci M, Giannoni E, Fearns A, Ribas R, Gao Q, Taddei ML (2016). miR-155 drives metabolic reprogramming of ER+ breast cancer cells following long-term estrogen deprivation and predicts clinical response to aromatase inhibitors. Cancer Res..

[47] Ahmad A, Aboukameel A, Kong D, Wang Z, Sethi S, Chen W (2011). Phosphoglucose isomerase/autocrine motility factor mediates epithelial-mesenchymal transition regulated by miR-200 in breast cancer cells. Cancer Res..

[48] Zhang J, Wang J, Xing H, Li Q, Zhao Q, Li J (2016). Down-regulation of FBP1 by ZEB1-mediated repression confers to growth and invasion in lung cancer cells. Mol. Cell Biochem.

[49] Li B, Qiu B, Lee DS, Walton ZE, Ochocki JD, Mathew LK (2014). Fructose-1,6-bisphosphatase opposes renal carcinoma progression. Nature.

[50] Masin M, Vazquez J, Rossi S, Groeneveld S, Samson N, Schwalie PC (2014). GLUT3 is induced during epithelial-mesenchymal transition and promotes tumor cell proliferation in non-small cell lung cancer. Cancer Metab..

[51] Ma Y, Wang W, Idowu MO, Oh U, Wang XY, Temkin SM (2018). Ovarian cancer relies on glucose transporter 1 to fuel glycolysis and growth: anti-tumor activity of BAY-876. Cancers (Basel).

[52] Zhang F, Lin JD, Zuo XY, Zhuang YX, Hong CQ, Zhang GJ (2017). Elevated transcriptional levels of aldolase A (ALDOA) associates with cell cycle-related genes in patients with NSCLC and several solid tumors. BioData Min..

[53] Gibadulinova A, Tothova V, Pastorek J, Pastorekova S (2011). Transcriptional regulation and functional implication of S100P in cancer. Amino Acids.

[54] Parkkila S, Pan PW, Ward A, Gibadulinova A, Oveckova I, Pastorekova S (2008). The calcium-binding protein S100P in normal and malignant human tissues. BMC Clin. Pathol..

[55] Sato N, Hitomi J (2002). S100P expression in human esophageal epithelial cells: human esophageal epithelial cells sequentially produce different S100 proteins in the process of differentiation. Anat. Rec..

[56] Guerreiro DSI, Hu YF, Russo IH, Ao X, Salicioni AM, Yang X (2000). S100P calcium-binding protein overexpression is associated with immortalization of human breast epithelial cells in vitro and early stages of breast cancer development in vivo. Int. J. Oncol..

[57] Hsieh HL, Schafer BW, Sasaki N, Heizmann CW (2003). Expression analysis of S100 proteins and RAGE in human tumors using tissue microarrays. Biochem. Biophys. Res. Commun..

[58] Koltzscher M, Neumann C, Konig S, Gerke V (2003). Ca2+-dependent binding and activation of dormant ezrin by dimeric S100P. Mol. Biol. Cell.

[59] Dowen SE, Crnogorac-Jurcevic T, Gangeswaran R, Hansen M, Eloranta JJ, Bhakta V (2005). Expression of S100P and its novel binding partner S100PBPR in early pancreatic cancer. Am. J. Pathol..

[60] Liu Y, Zhang X, Yang B, Zhuang H, Guo H, Wei W (2018). Demethylation-induced overexpression of Shc3 drives c-Raf-independent activation of MEK/ERK in HCC. Cancer Res..

[61] Honda T, Tamura G, Waki T, Kawata S, Terashima M, Nishizuka S (2004). Demethylation of MAGE promoters during gastric cancer progression. Br. J. Cancer.

[62] Wu SC, Zhang Y (2010). Active DNA demethylation: many roads lead to Rome. Nat. Rev. Mol. Cell Biol..

[63] Ye Q, Zheng MH, Cai Q, Feng B, Chen XH, Yu BQ (2008). Aberrant expression and demethylation of gamma-synuclein in colorectal cancer, correlated with progression of the disease. Cancer Sci..

