# Supplementary material for: S100P contributes to promoter demethylation and transcriptional activation of SLC2A5 to promote metastasis in colorectal cancer
Source: Br J Cancer. 2021 Jun 29;125(5):734–47. doi: 10.1038/s41416-021-01306-z (PMC8405647; doi:10.1038/s41416-021-01306-z)
Supplement: Supplementary file 1 — Supplementary Materials [file 41416_2021_1306_MOESM1_ESM.docx]

**Supplementary Tables**

**Supplementary Table 1. Primer and shRNA sequences used in this study**

| **Primer name** | **Forward** | **Reverse** | **Experiment** |
| --- | --- | --- | --- |
| GAPDH | GCACCGTCAAGGCTGAGAAC | TGGTGAAGACGCCAGTGGA | qRT-PCR |
| SLC2A5 | CGTGCCTGCGATCTTAATGG | GATACACCTGCACATATTCCCAC | qRT-PCR |
| S100P | TGCAGAGTGGAAAAGACAAGGAT | CCACCTGGGCATCTCCATT | qRT-PCR |
| SLC2A5-A | ATCGTTGATTTCCTCTCCCCACC | AACGCATAGCAAGACCAGAGGT | ChIP-qPCR |
| SLC2A5-B | CAGGCGTGAGCCACCGC | GAGACGGGTACAGGTGTGCAATC | ChIP-qPCR |
| SLC2A5-C | CCCGCTTCCCCGGGCTG | GGCGAGGGGGTAACTAAAACCG | ChIP-qPCR |
| SLC2A5-D | CGCCATGGCTGTGCTTGC | ATCCCGGGAGCTGCGGG | ChIP-qPCR |
| SLC2A5-E | CGGCCCGCAGCCTGGCG | TCACCAACGCTCGACCCGT | ChIP-qPCR |
| MSP-M | GGGATAGGATTTATTCGGAGTTC | CGATTCCTACCAATAACAAAACG | PCR |
| MSP-U | AGGGATAGGATTTATTTGGAGTTTG | CCAATTCCTACCAATAACAAAACAT | PCR |
| BSP | GAGCGTTGGTGACGTTATGGTTGTGTTTG | ACCCAAATCTTCCACTTCTCCCTACCTT | PCR |
| SLC2A5 promoter PCR | GATGCATGAGATTCAGAATC | CTTTATGTTTTTGGCGTCTTCCA | Luc vectors construction |
| **List of shRNA coding sequences** | | | |
|  | Target sequence | | |
| SLC2A5 shRNA | CAGATCTTTGGTCTTCGGAAT | | |
| S100P shRNA | CCGUGGAUAAAUUGCUCAATT | | |

**Supplementary Table 2. Target genes obtained by ChIP-sequencing of nuclear S100P**

| **Chr** | **Start** | **End** | **Annotation** | **Distance to TSS** | **Gene** | **Fold_enrichment** |
| --- | --- | --- | --- | --- | --- | --- |
| chr1 | 9131610 | 9131892 | CpG | -1864 | SLC2A5 | 27.21 |
| chr1 | 212780086 | 212780242 | CpG | -1806 | ATF3 | 24.42 |
| chr1 | 55462674 | 55463042 | CpG | -1759 | BSND | 19.84 |
| chr1 | 212780248 | 212780357 | CpG | -1668 | ATF3 | 19.36 |
| chr1 | 91184190 | 91184615 | CpG | -1608 | BARHL2 | 19.16 |
| chr1 | 181451280 | 181451664 | CpG | -1214 | CACNA1E | 18.04 |
| chr1 | 85463776 | 85464059 | CpG | -1121 | MCOLN2 | 17.64 |
| chr10 | 104402210 | 104402561 | CpG | -1867 | TRIM8 | 17.64 |
| chr10 | 33624986 | 33625244 | CpG-2701 | -1282 | NRP1 | 17.42 |
| chr10 | 135050097 | 135050283 | CpG | -1218 | VENTX | 17.32 |
| chr11 | 43600805 | 43601120 | CpG | -1982 | MIR129-2 | 17.25 |
| chr11 | 17755982 | 17756335 | CpG | -1337 | KCNC1 | 17.09 |
| chr11 | 57226673 | 57226948 | CpG | -1200 | RTN4RL2 | 15.87 |
| chr11 | 134282685 | 134283081 | CpG-4966 | -1071 | B3GAT1 | 15.87 |
| chr11 | 20619464 | 20620325 | CpG | -1052 | SLC6A5 | 15.87 |
| chr12 | 12877167 | 12877443 | CpG | -1546 | APOLD1 | 15.49 |
| chr12 | 131647725 | 131648441 | CpG | -1473 | RP11-638F5.1 | 15.26 |
| chr12 | 133065775 | 133066102 | CpG | -1219 | FBRSL1 | 15.2 |
| chr12 | 49931632 | 49932113 | CpG | -1068 | KCNH3 | 15.12 |
| chr13 | 108520687 | 108521029 | CpG | -1398 | FAM155A | 15.04 |
| chr13 | 24882269 | 24882554 | CpG | -1305 | C1QTNF9 | 14.97 |
| chr14 | 69950341 | 69950580 | CpG | -1011 | PLEKHD1 | 14.97 |
| chr15 | 74044729 | 74045169 | CpG | -1133 | C15orf59 | 14.43 |
| chr16 | 3017226 | 3017798 | CpG-8725 | -1734 | PAQR4 | 14.06 |
| chr16 | 65157268 | 65157630 | CpG-9360 | -1530 | CDH11 | 13.92 |
| chr16 | 14396264 | 14396535 | CpG | -1425 | MIR193B | 13.68 |
| chr16 | 86599231 | 86599742 | CpG | -1371 | FOXC2 | 13.68 |
| chr16 | 51186035 | 51186621 | CpG-9270 | -1145 | SALL1 | 13.61 |
| chr17 | 59531507 | 59532372 | CpG | -1868 | TBX4 | 13.55 |
| chr17 | 47073123 | 47073450 | CpG | -1488 | IGF2BP1 | 13.48 |
| chr17 | 45771026 | 45771396 | CpG | -1419 | TBKBP1 | 13.23 |
| chr17 | 77771831 | 77772795 | CpG-11232 | -1398 | CBX8 | 13.23 |
| chr17 | 48350473 | 48350674 | CpG | -1215 | TMEM92 | 13.09 |
| chr17 | 71641226 | 71641623 | CpG-11063 | -1197 | SDK2 | 13.07 |
| chr17 | 78450648 | 78452555 | CpG-11274 | -1197 | NPTX1 | 12.96 |
| chr17 | 66193387 | 66193975 | CpG | -1120 | LOC440461 | 12.53 |
| chr18 | 5630080 | 5630833 | CpG-11574 | -1466 | EPB41L3 | 12.35 |
| chr18 | 35146913 | 35147644 | CpG-11742 | -1278 | CELF4 | 12.21 |
| chr19 | 42827998 | 42828827 | CpG | -1349 | MEGF8 | 12.03 |
| chr19 | 47523003 | 47523204 | CpG | -1040 | NPAS1 | 11.27 |
| chr2 | 121101767 | 121102194 | CpG | -1739 | INHBB | 11.14 |
| chr2 | 237476506 | 237476795 | CpG | -1730 | ACKR3 | 10.95 |
| chr2 | 26401756 | 26401989 | CpG | -1713 | GAREML | 10.58 |
| chr2 | 5830939 | 5831482 | CpG | -1589 | SOX11 | 10.58 |
| chr2 | 289635 | 290102 | CpG-14598 | -1560 | FAM150B | 10.48 |
| chr2 | 190538983 | 190539545 | CpG | -1447 | ANKAR | 10.2 |
| chr2 | 128786009 | 128786319 | CpG-15506 | -1295 | SAP130 | 10.16 |
| chr2 | 47798367 | 47798866 | CpG-14960 | -1146 | KCNK12 | 10.16 |
| chr20 | 61808353 | 61808603 | CpG | -1374 | MIR124-3 | 10.08 |
| chr20 | 57224978 | 57225215 | CpG | -1213 | STX16 | 10.05 |
| chr21 | 45662512 | 45663084 | CpG-17318 | -1911 | ICOSLG | 9.59 |
| chr22 | 29467444 | 29467652 | CpG | -1518 | KREMEN1 | 9.38 |
| chr22 | 35936185 | 35936410 | CpG | -1055 | RASD2 | 9.34 |
| chr3 | 96531907 | 96532664 | CpG | -1140 | EPHA6 | 9.34 |
| chr3 | 54155548 | 54155750 | CpG | -1044 | CACNA2D3 | 9.23 |
| chr4 | 149365379 | 149365790 | CpG-20176 | -1912 | NR3C2 | 9.18 |
| chr4 | 90031957 | 90032292 | CpG | -1844 | TIGD2 | 8.98 |
| chr4 | 2042073 | 2042538 | CpG | -1415 | C4orf48 | 8.64 |
| chr4 | 81104901 | 81105157 | CpG | -1395 | PRDM8 | 8.58 |
| chr4 | 330202 | 330744 | CpG | -1123 | ZNF141 | 8.44 |
| chr5 | 131991941 | 131992444 | CpG | -1673 | IL13 | 8.23 |
| chr5 | 83017793 | 83018175 | CpG | -1088 | HAPLN1 | 8.19 |
| chr6 | 99280369 | 99281077 | CpG | -1857 | POU3F2 | 8.11 |
| chr6 | 126068736 | 126069318 | CpG | -1705 | HEY2 | 7.9 |
| chr6 | 100912960 | 100913386 | CpG | -1622 | SIM1 | 7.81 |
| chr6 | 25139973 | 25140072 | CpG | -1402 | CMAHP | 7.77 |
| chr6 | 168378716 | 168378950 | CpG | -1214 | HGC6.3 | 7.6 |
| chr7 | 39015488 | 39016031 | CpG | -1850 | POU6F2 | 7.51 |
| chr7 | 127672539 | 127672950 | CpG | -1742 | LRRC4 | 7.46 |
| chr7 | 113724886 | 113725262 | CpG | -1291 | FOXP2 | 7.22 |
| chr7 | 100845178 | 100845681 | CpG | -1127 | MOGAT3 | 7.08 |
| chr7 | 127671903 | 127672260 | CpG | -1079 | LRRC4 | 6.01 |
| chr8 | 26724205 | 26724682 | CpG | -1521 | ADRA1A | 5.99 |
| chr8 | 132053781 | 132054493 | CpG | -1302 | ADCY8 | 5.97 |
| chr8 | 56013671 | 56014132 | CpG | -1116 | XKR4 | 5.88 |
| chr8 | 25903565 | 25903836 | CpG-25528 | -1060 | EBF2 | 5.38 |
| chr9 | 19788651 | 19789333 | CpG | -1975 | SLC24A2 | 5.12 |
| chr9 | 95569989 | 95570406 | CpG | -1696 | ANKRD19P | 4.76 |
| chr9 | 139294136 | 139294566 | CpG | -1462 | SNAPC4 | 4.67 |
| chr9 | 68455673 | 68455907 | CpG | -1415 | LOC642236 | 4.56 |

**Supplementary Figures**

**Supplementary Figure S1**

**
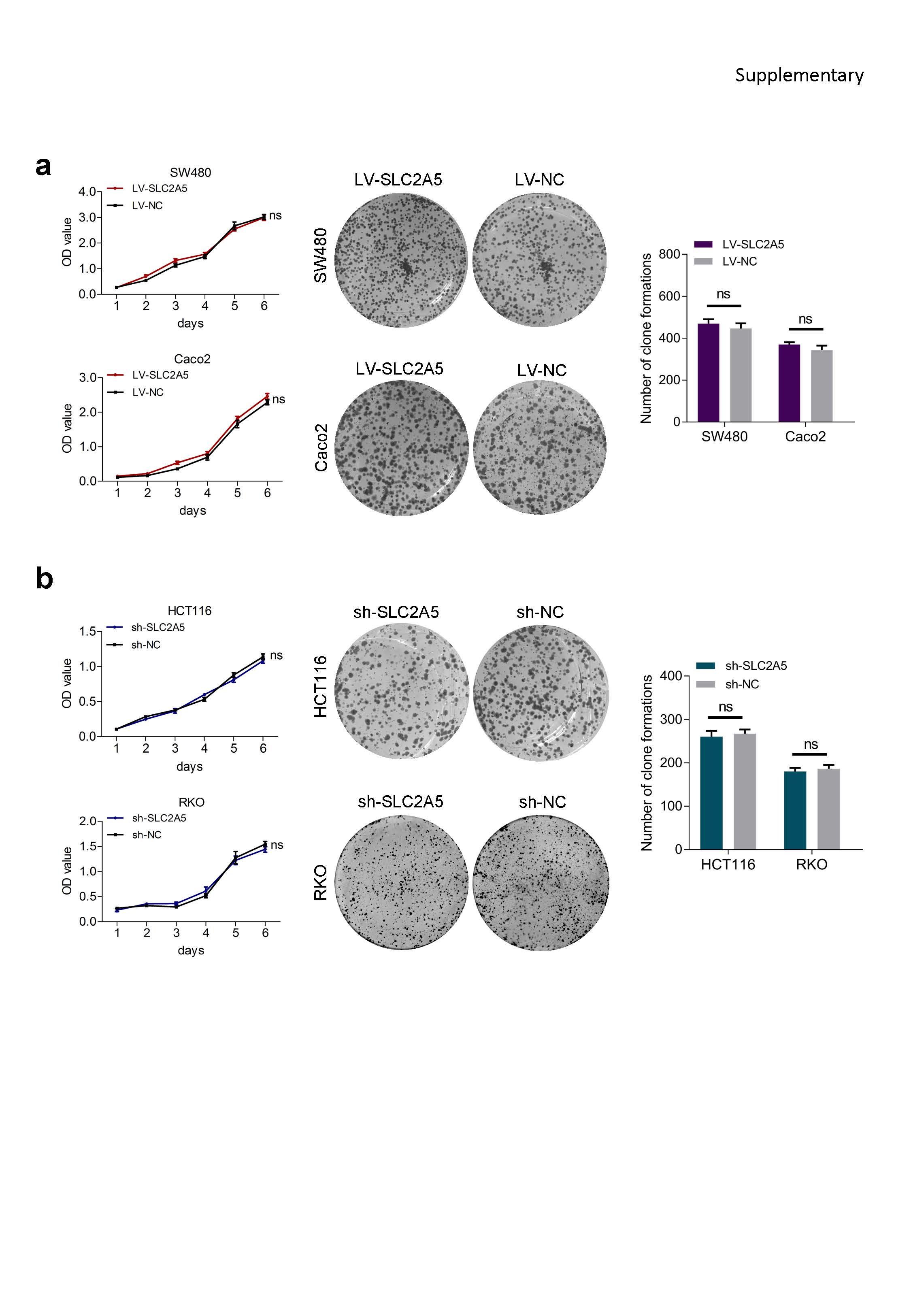
**

**Supplementary Figure S1. SLC2A5 has no significant effect on CRC cell proliferation. (a)** CCK8 and colony formation assays of SW480 and Caco2 cells with LV-SLC2A5 or LV-NC. Results are presented as mean ± SEM (n=3), based on Student’s t-test. **(b)** CCK8 and colony formation assay of HCT116 and RKO cells with sh-SLC2A5 or sh-NC. Results are presented as mean ± SEM (n=3), based on Student’s t-test.

**Supplementary Figure S2**

**
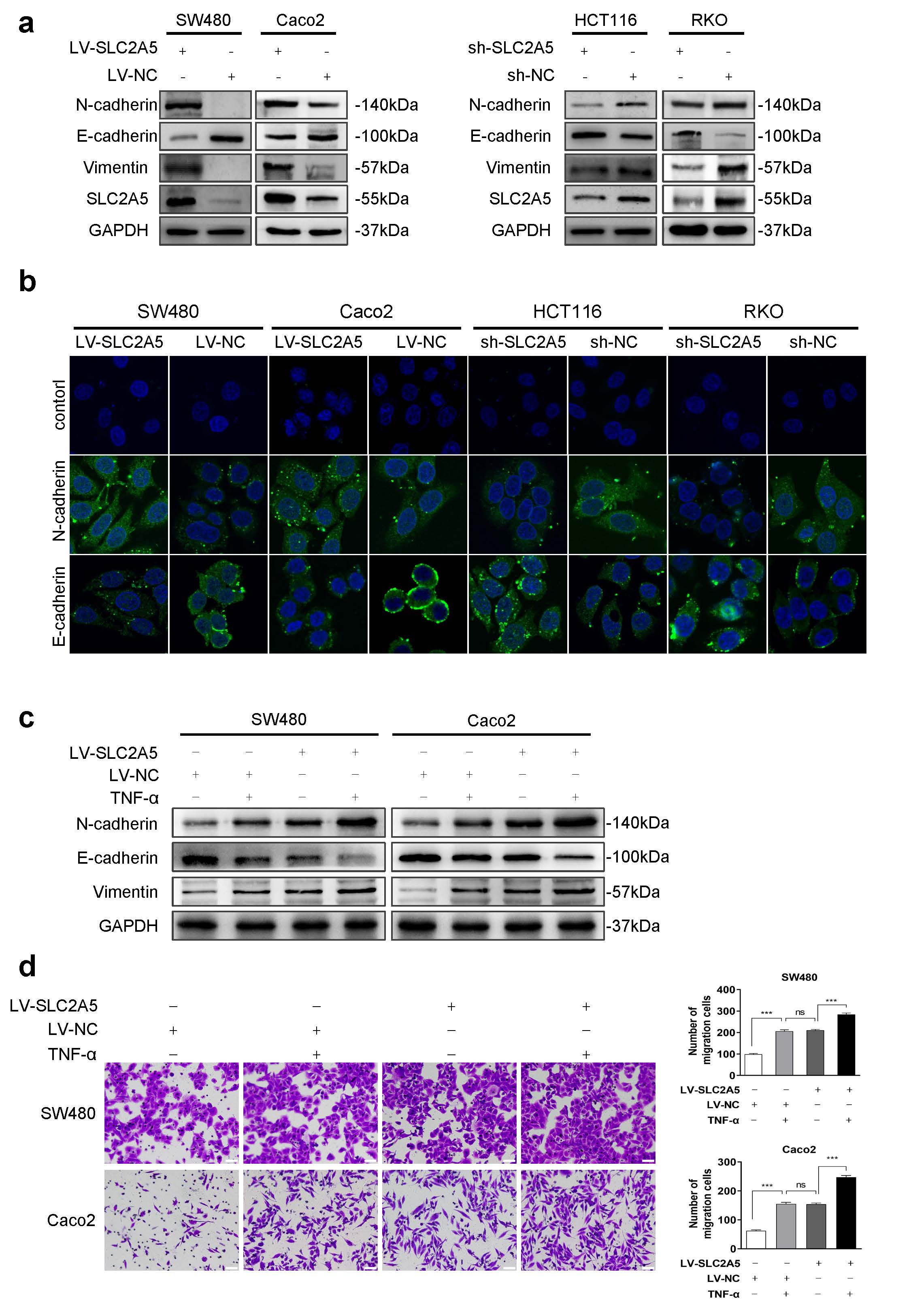
**

**Supplementary Figure S2.** **Western blotting analysis of EMT-related markers in CRC cell lines with different SLC2A5 expression.** Protein expression levels of E-cadherin, N-cadherin and Vimentin in indicated cell lines with different SLC2A5 expression. GAPDH was loaded as a control.

**Supplementary Figure S3**


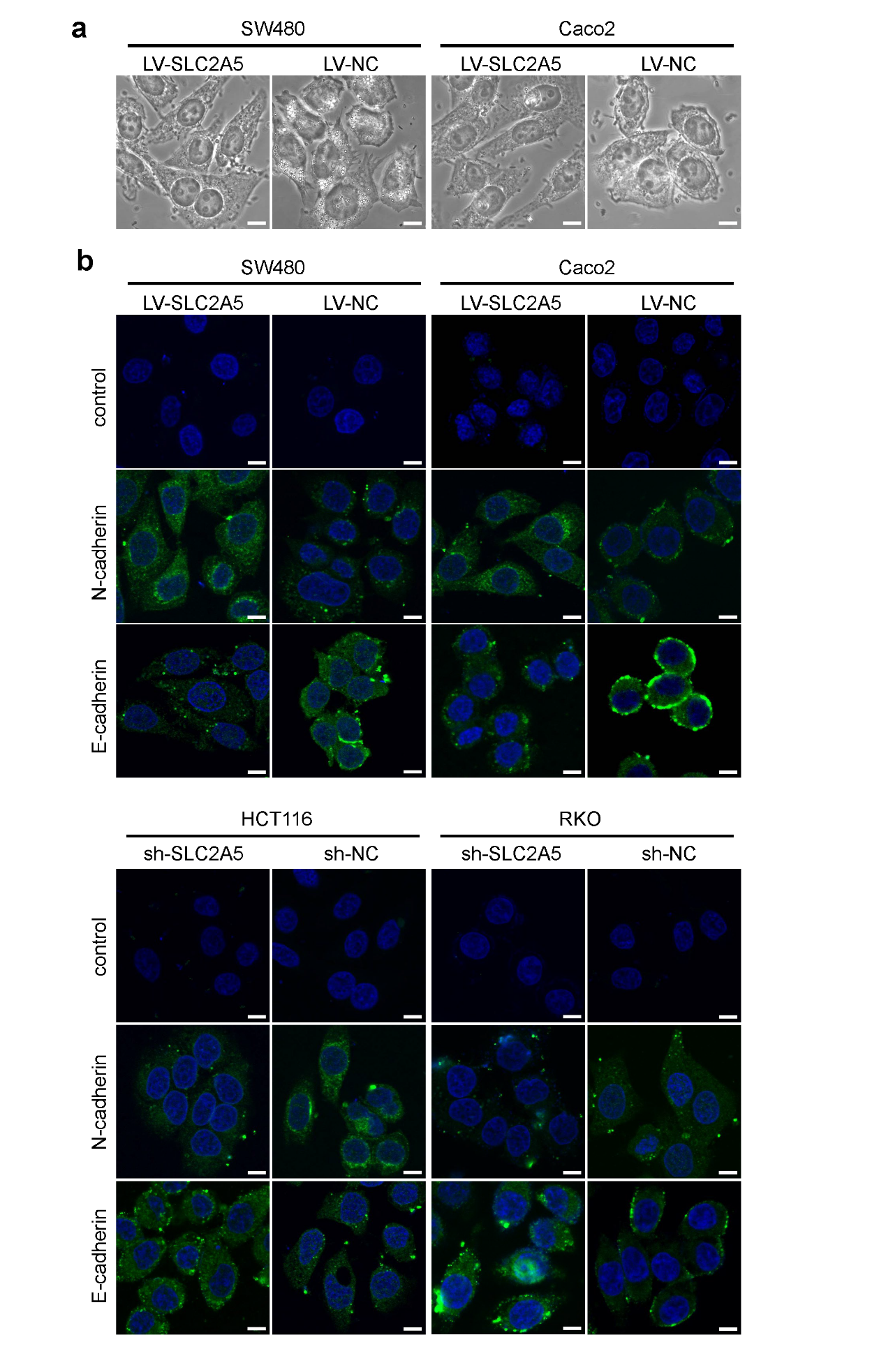


**Supplementary Figure S3. SLC2A5 partially stimulates EMT in CRC cells. (a)** Phase contrast images of SW480 and Caco2 cells overexpressing SLC2A5 (LV-SLC2A5) or vector (LV-NC). Scale bar, 10μm (60×). **(b)** IF analysis of N-cadherin and E-cadherin in indicated cell lines with different SLC2A5 expression. The control was treated with PBS instead of primary antibody. Scale bar, 10μm (60×).

**Supplementary Figure S4**

**
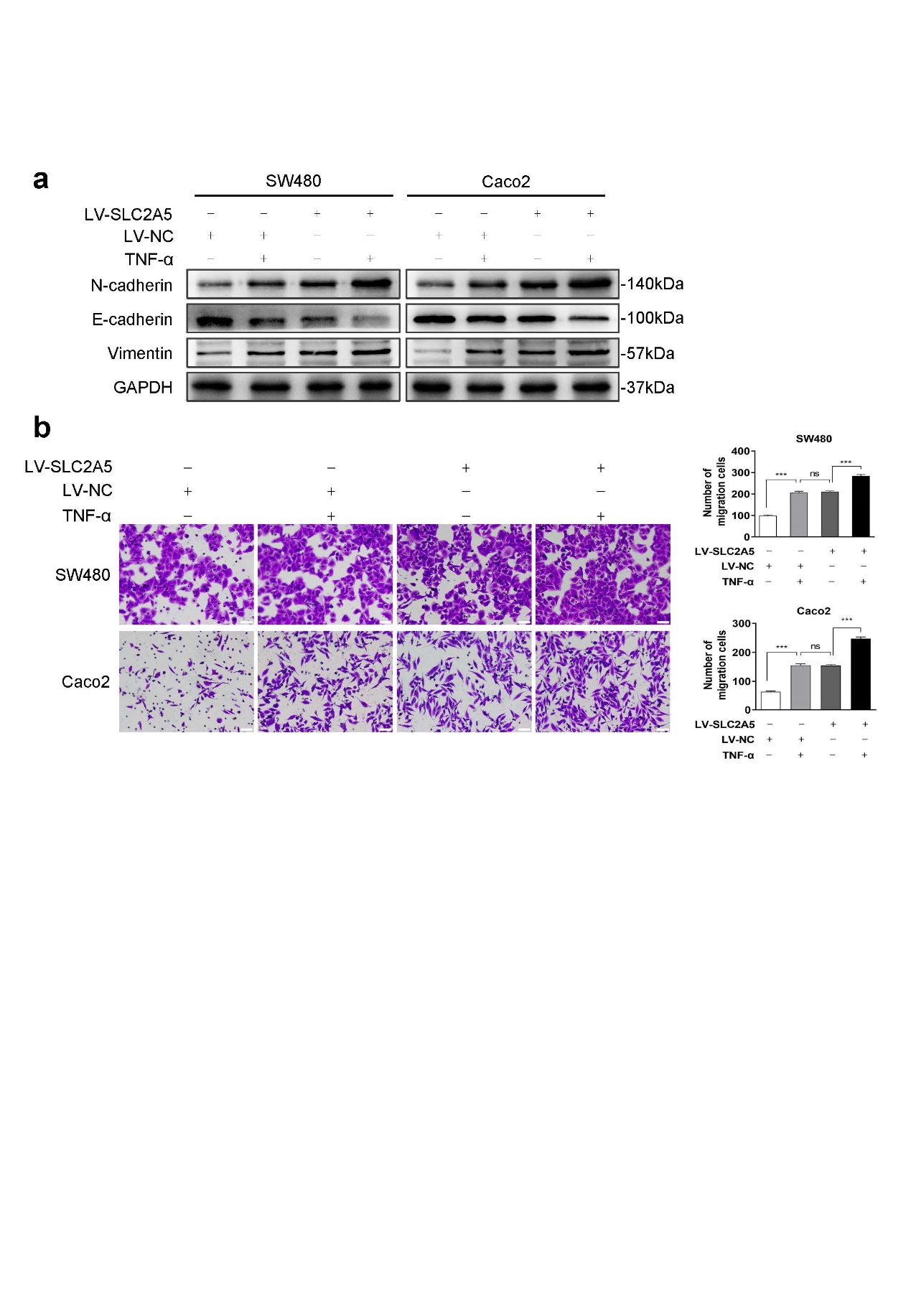
**

**Supplementary Figure S4. EMT is essential for SLC2A5-mediated CRC cell migration.** SW480 and Caco2 cells with SLC2A5 overexpression (LV-SLC2A5) or vector (LV-NC) were treated with TNF-α 50ng/ml, ddH_2_O containing 1‰ BSA was used as a control. **(a)** Protein expression levels of N-cadherin, E-cadherin and Vimentin were detected using western blotting. **(b)** Migration ability of indicated cells were detected using transwell assays. Scale bar, 50μm (20×). Results are presented as mean ± SEM (n=3). ^***^*P* < 0.001, based on Student’s t-test.

**Supplementary Figure S5**

**
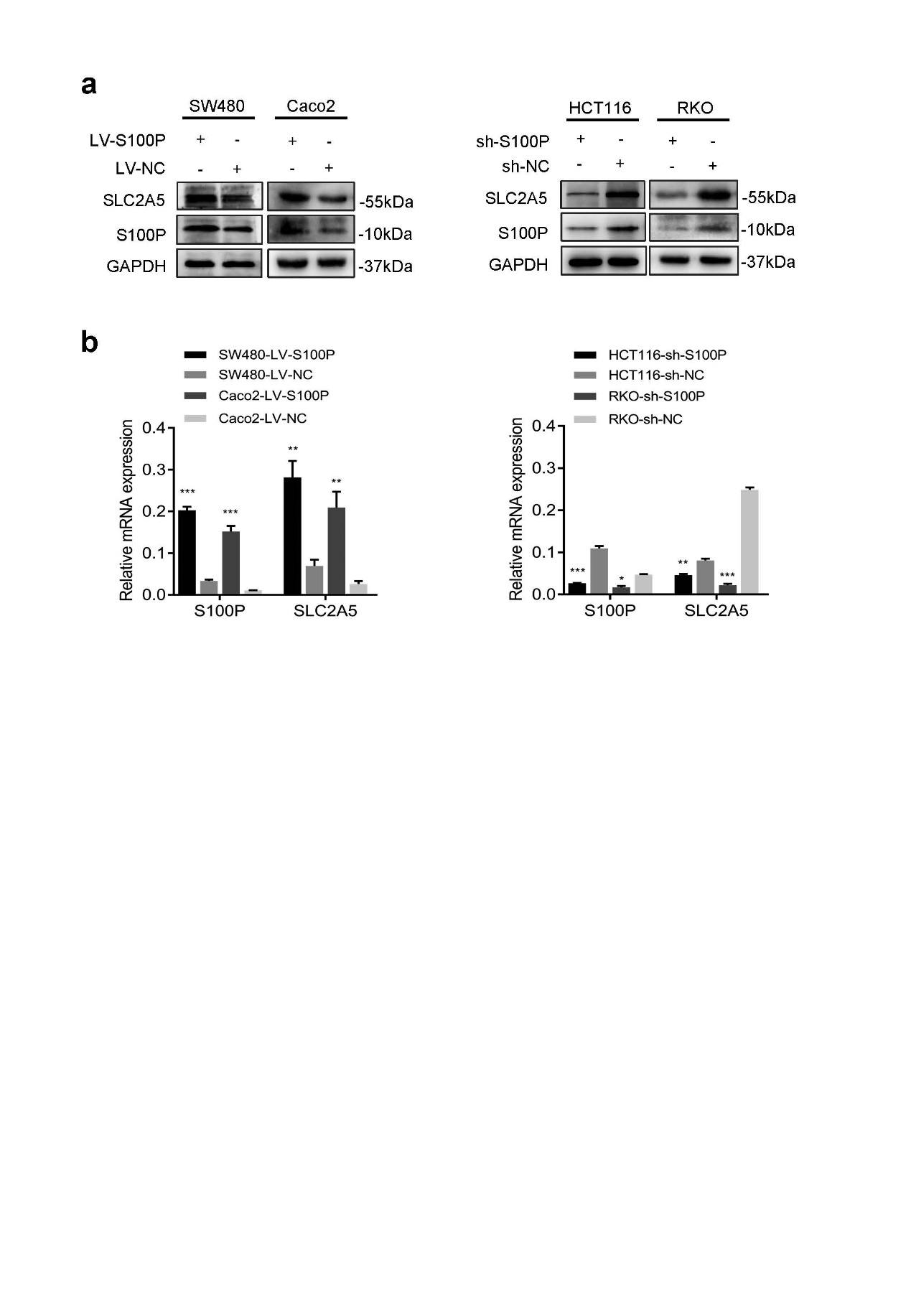
**

**Supplementary Figure S5.** **The expression level of SLC2A5 is regulated by S100P.** **(a, b)** Protein and mRNA expression levels of SLC2A5 in indicated cell lines with different S100P expression by western blotting **(a)** and qRT-PCR **(b)**. GAPDH was loaded as a control. Results are shown as mean ± SEM (n=3). ^*^*P* < 0.05, ^**^*P* < 0.01, ^***^*P* < 0.001, based on Student’s t-test.

**Supplementary Figure S6**


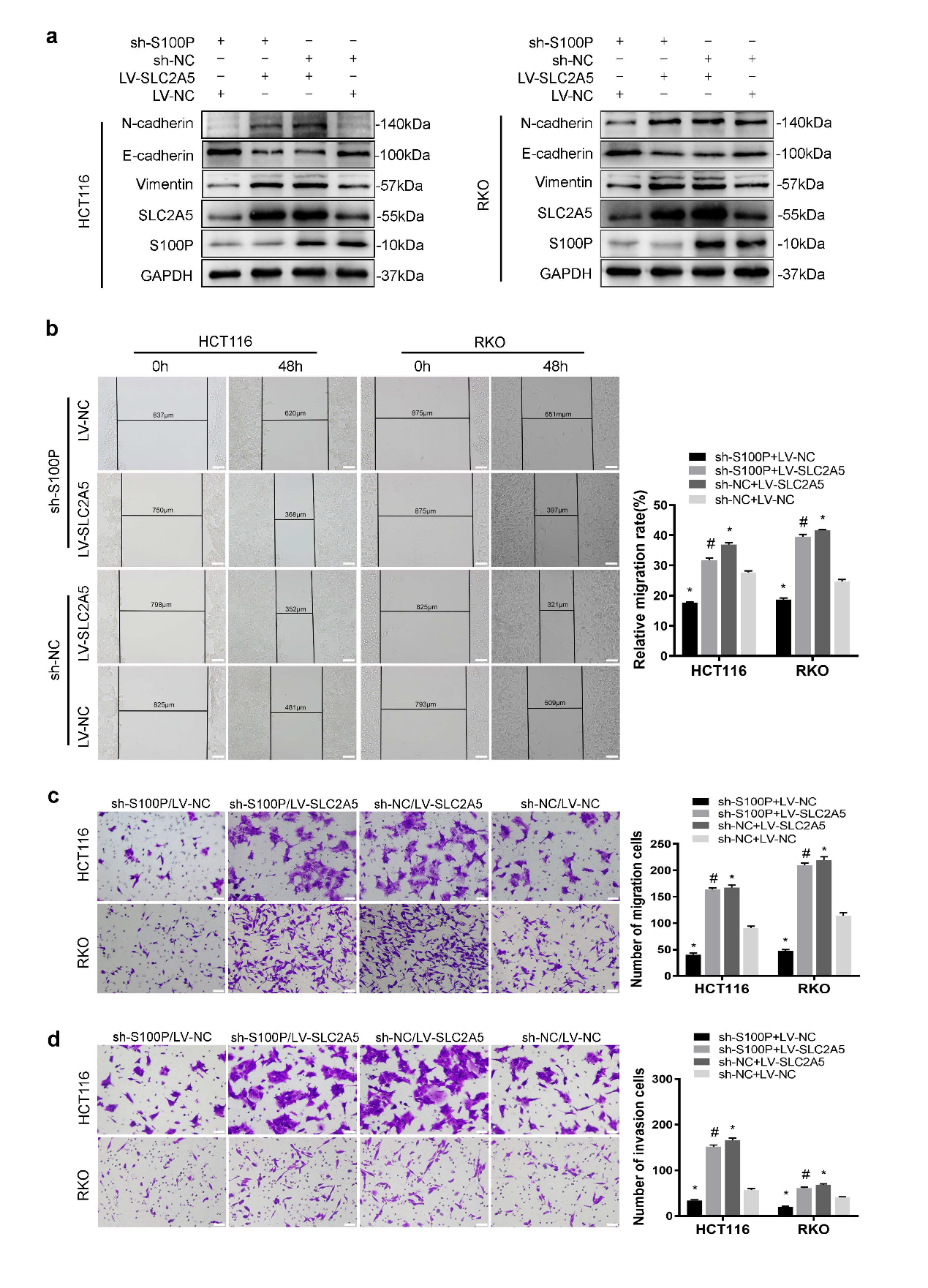


**Supplementary Figure S6.** **SLC2A5 restoration enhances the invasion and migration abilities in S100P knockdown cells.** SLC2A5 overexpressed lentivirus (LV-SLC2A5) or vector (LV-NC) was transfected in HCT116 and RKO cells that expressed S100P shRNA (sh-S100P) or vector (sh-NC). **(a)** Protein expression levels of N-cadherin, E-cadherin, Vimentin, SLC2A5, S100P and GAPDH were detected using western blotting. (**b-d)** The migration and invasion abilities were measured by wound healing assays (10×) **(b)** and transwell assays (20×) without **(c)** or with **(d)** Matrigel. Scale bar, 100μm (10×), 50μm (20×). Results are shown as mean ± SEM (n=3). ^*^*P* < 0.01, compared to sh-NC+ LV-NC; ^#^*P* < 0.01, compared to sh-S100P + LV-NC. Student’s t-test were used to analyze the data.
